# Measurements of ${\mathrm{p}} {\mathrm{p}} \rightarrow {\mathrm{Z}} {\mathrm{Z}}$ production cross sections and constraints on anomalous triple gauge couplings at $\sqrt{s} = 13\,\text {TeV}$

**DOI:** 10.1140/epjc/s10052-020-08817-8

**Published:** 2021-03-01

**Authors:** A. M. Sirunyan, A. Tumasyan, W. Adam, F. Ambrogi, T. Bergauer, M. Dragicevic, J. Erö, A. Escalante Del Valle, R. Frühwirth, M. Jeitler, N. Krammer, L. Lechner, D. Liko, T. Madlener, I. Mikulec, F. M. Pitters, N. Rad, J. Schieck, R. Schöfbeck, M. Spanring, S. Templ, W. Waltenberger, C.-E. Wulz, M. Zarucki, V. Chekhovsky, A. Litomin, V. Makarenko, J. Suarez Gonzalez, M. R. Darwish, E. A. De Wolf, D. Di Croce, X. Janssen, T. Kello, A. Lelek, M. Pieters, H. Rejeb Sfar, H. Van Haevermaet, P. Van Mechelen, S. Van Putte, N. Van Remortel, F. Blekman, E. S. Bols, S. S. Chhibra, J. D’Hondt, J. De Clercq, D. Lontkovskyi, S. Lowette, I. Marchesini, S. Moortgat, A. Morton, Q. Python, S. Tavernier, W. Van Doninck, P. Van Mulders, D. Beghin, B. Bilin, B. Clerbaux, G. De Lentdecker, B. Dorney, L. Favart, A. Grebenyuk, A. K. Kalsi, I. Makarenko, L. Moureaux, L. Pétré, A. Popov, N. Postiau, E. Starling, L. Thomas, C. Vander Velde, P. Vanlaer, D. Vannerom, L. Wezenbeek, T. Cornelis, D. Dobur, M. Gruchala, I. Khvastunov, M. Niedziela, C. Roskas, K. Skovpen, M. Tytgat, W. Verbeke, B. Vermassen, M. Vit, G. Bruno, F. Bury, C. Caputo, P. David, C. Delaere, M. Delcourt, I. S. Donertas, A. Giammanco, V. Lemaitre, K. Mondal, J. Prisciandaro, A. Taliercio, M. Teklishyn, P. Vischia, S. Wuyckens, J. Zobec, G. A. Alves, G. Correia Silva, C. Hensel, A. Moraes, W. L. Aldá Júnior, E. Belchior Batista Das Chagas, H. BRANDAO MALBOUISSON, W. Carvalho, J. Chinellato, E. Coelho, E. M. Da Costa, G. G. Da Silveira, D. De Jesus Damiao, S. Fonseca De Souza, J. Martins, D. Matos Figueiredo, M. Medina Jaime, M. Melo De Almeida, C. Mora Herrera, L. Mundim, H. Nogima, P. Rebello Teles, L. J. Sanchez Rosas, A. Santoro, S. M. Silva Do Amaral, A. Sznajder, M. Thiel, E. J. Tonelli Manganote, F. Torres Da Silva De Araujo, A. Vilela Pereira, C. A. Bernardes, L. Calligaris, T. R. Fernandez Perez Tomei, E. M. Gregores, D. S. Lemos, P. G. Mercadante, S. F. Novaes, Sandra S. Padula, A. Aleksandrov, G. Antchev, I. Atanasov, R. Hadjiiska, P. Iaydjiev, M. Misheva, M. Rodozov, M. Shopova, G. Sultanov, M. Bonchev, A. Dimitrov, T. Ivanov, L. Litov, B. Pavlov, P. Petkov, A. Petrov, W. Fang, Q. Guo, H. Wang, L. Yuan, M. Ahmad, Z. Hu, Y. Wang, E. Chapon, G. M. Chen, H. S. Chen, M. Chen, T. Javaid, A. Kapoor, D. Leggat, H. Liao, Z. Liu, R. Sharma, A. Spiezia, J. Tao, J. Thomas-wilsker, J. Wang, H. Zhang, J. Zhao, A. Agapitos, Y. Ban, C. Chen, Q. Huang, A. Levin, Q. Li, M. Lu, X. Lyu, Y. Mao, S. J. Qian, D. Wang, Q. Wang, J. Xiao, Z. You, X. Gao, M. Xiao, C. Avila, A. Cabrera, C. Florez, J. Fraga, A. Sarkar, M. A. Segura Delgado, J. Jaramillo, J. Mejia Guisao, F. Ramirez, J. D. Ruiz Alvarez, C. A. Salazar González, N. Vanegas Arbelaez, D. Giljanovic, N. Godinovic, D. Lelas, I. Puljak, T. Sculac, Z. Antunovic, M. Kovac, V. Brigljevic, D. Ferencek, D. Majumder, M. Roguljic, A. Starodumov, T. Susa, M. W. Ather, A. Attikis, E. Erodotou, A. Ioannou, G. Kole, M. Kolosova, S. Konstantinou, G. Mavromanolakis, J. Mousa, C. Nicolaou, F. Ptochos, P. A. Razis, H. Rykaczewski, H. Saka, D. Tsiakkouri, M. Finger, M. Finger, A. Kveton, J. Tomsa, E. Ayala, E. Carrera Jarrin, H. Abdalla, Y. Assran, A. Mohamed, A. Lotfy, M. A. Mahmoud, S. Bhowmik, A. Carvalho Antunes De Oliveira, R. K. Dewanjee, K. Ehataht, M. Kadastik, M. Raidal, C. Veelken, P. Eerola, L. Forthomme, H. Kirschenmann, K. Osterberg, M. Voutilainen, E. Brücken, F. Garcia, J. Havukainen, V. Karimäki, M. S. Kim, R. Kinnunen, T. Lampén, K. Lassila-Perini, S. Laurila, S. Lehti, T. Lindén, H. Siikonen, E. Tuominen, J. Tuominiemi, P. Luukka, T. Tuuva, C. Amendola, M. Besancon, F. Couderc, M. Dejardin, D. Denegri, J. L. Faure, F. Ferri, S. Ganjour, A. Givernaud, P. Gras, G. Hamel de Monchenault, P. Jarry, B. Lenzi, E. Locci, J. Malcles, J. Rander, A. Rosowsky, M.Ö. Sahin, A. Savoy-Navarro, M. Titov, G. B. Yu, S. Ahuja, F. Beaudette, M. Bonanomi, A. Buchot Perraguin, P. Busson, C. Charlot, O. Davignon, B. Diab, G. Falmagne, R. Granier de Cassagnac, A. Hakimi, I. Kucher, A. Lobanov, C. Martin Perez, M. Nguyen, C. Ochando, P. Paganini, J. Rembser, R. Salerno, J. B. Sauvan, Y. Sirois, A. Zabi, A. Zghiche, J.-L. Agram, J. Andrea, D. Bloch, G. Bourgatte, J.-M. Brom, E. C. Chabert, C. Collard, J.-C. Fontaine, D. Gelé, U. Goerlach, C. Grimault, A.-C. Le Bihan, P. Van Hove, E. Asilar, S. Beauceron, C. Bernet, G. Boudoul, C. Camen, A. Carle, N. Chanon, D. Contardo, P. Depasse, H. El Mamouni, J. Fay, S. Gascon, M. Gouzevitch, B. Ille, Sa. Jain, I. B. Laktineh, H. Lattaud, A. Lesauvage, M. Lethuillier, L. Mirabito, L. Torterotot, G. Touquet, M. Vander Donckt, S. Viret, T. Toriashvili, Z. Tsamalaidze, L. Feld, K. Klein, M. Lipinski, D. Meuser, A. Pauls, M. Preuten, M. P. Rauch, J. Schulz, M. Teroerde, D. Eliseev, M. Erdmann, P. Fackeldey, B. Fischer, S. Ghosh, T. Hebbeker, K. Hoepfner, H. Keller, L. Mastrolorenzo, M. Merschmeyer, A. Meyer, P. Millet, G. Mocellin, S. Mondal, S. Mukherjee, D. Noll, A. Novak, T. Pook, A. Pozdnyakov, T. Quast, M. Radziej, Y. Rath, H. Reithler, J. Roemer, A. Schmidt, S. C. Schuler, A. Sharma, S. Wiedenbeck, S. Zaleski, C. Dziwok, G. Flügge, W. Haj Ahmad, O. Hlushchenko, T. Kress, A. Nowack, C. Pistone, O. Pooth, D. Roy, H. Sert, A. Stahl, T. Ziemons, H. Aarup Petersen, M. Aldaya Martin, P. Asmuss, I. Babounikau, S. Baxter, O. Behnke, A. Bermúdez Martínez, A. A. Bin Anuar, K. Borras, V. Botta, D. Brunner, A. Campbell, A. Cardini, P. Connor, S. Consuegra Rodríguez, V. Danilov, A. De Wit, M. M. Defranchis, L. Didukh, D. Domínguez Damiani, G. Eckerlin, D. Eckstein, T. Eichhorn, L. I. Estevez Banos, E. Gallo, A. Geiser, A. Giraldi, A. Grohsjean, M. Guthoff, A. Harb, A. Jafari, N. Z. Jomhari, H. Jung, A. Kasem, M. Kasemann, H. Kaveh, C. Kleinwort, J. Knolle, D. Krücker, W. Lange, T. Lenz, J. Lidrych, K. Lipka, W. Lohmann, R. Mankel, I.-A. Melzer-Pellmann, J. Metwally, A. B. Meyer, M. Meyer, M. Missiroli, J. Mnich, A. Mussgiller, V. Myronenko, Y. Otarid, D. Pérez Adán, S. K. Pflitsch, D. Pitzl, A. Raspereza, A. Saggio, A. Saibel, M. Savitskyi, V. Scheurer, P. Schütze, C. Schwanenberger, A. Singh, R. E. Sosa Ricardo, N. Tonon, O. Turkot, A. Vagnerini, M. Van De Klundert, R. Walsh, D. Walter, Y. Wen, K. Wichmann, C. Wissing, S. Wuchterl, O. Zenaiev, R. Zlebcik, R. Aggleton, S. Bein, L. Benato, A. Benecke, K. De Leo, T. Dreyer, A. Ebrahimi, M. Eich, F. Feindt, A. Fröhlich, C. Garbers, E. Garutti, P. Gunnellini, J. Haller, A. Hinzmann, A. Karavdina, G. Kasieczka, R. Klanner, R. Kogler, V. Kutzner, J. Lange, T. Lange, A. Malara, C. E. N. Niemeyer, A. Nigamova, K. J. Pena Rodriguez, O. Rieger, P. Schleper, S. Schumann, J. Schwandt, D. Schwarz, J. Sonneveld, H. Stadie, G. Steinbrück, B. Vormwald, I. Zoi, M. Baselga, S. Baur, J. Bechtel, T. Berger, E. Butz, R. Caspart, T. Chwalek, W. De Boer, A. Dierlamm, A. Droll, K. El Morabit, N. Faltermann, K. Flöh, M. Giffels, A. Gottmann, F. Hartmann, C. Heidecker, U. Husemann, M. A. Iqbal, I. Katkov, P. Keicher, R. Koppenhöfer, S. Maier, M. Metzler, S. Mitra, D. Müller, Th. Müller, M. Musich, G. Quast, K. Rabbertz, J. Rauser, D. Savoiu, D. Schäfer, M. Schnepf, M. Schröder, D. Seith, I. Shvetsov, H. J. Simonis, R. Ulrich, M. Wassmer, M. Weber, R. Wolf, S. Wozniewski, G. Anagnostou, P. Asenov, G. Daskalakis, T. Geralis, A. Kyriakis, D. Loukas, G. Paspalaki, A. Stakia, M. Diamantopoulou, D. Karasavvas, G. Karathanasis, P. Kontaxakis, C. K. Koraka, A. Manousakis-katsikakis, A. Panagiotou, I. Papavergou, N. Saoulidou, K. Theofilatos, K. Vellidis, E. Vourliotis, G. Bakas, K. Kousouris, I. Papakrivopoulos, G. Tsipolitis, A. Zacharopoulou, I. Evangelou, C. Foudas, P. Gianneios, P. Katsoulis, P. Kokkas, S. Mallios, K. Manitara, N. Manthos, I. Papadopoulos, J. Strologas, M. Bartók, R. Chudasama, M. Csanad, M. M. A. Gadallah, S. Lökös, P. Major, K. Mandal, A. Mehta, G. Pasztor, O. Surányi, G. I. Veres, G. Bencze, C. Hajdu, D. Horvath, F. Sikler, V. Veszpremi, G. Vesztergombi, S. Czellar, J. Karancsi, J. Molnar, Z. Szillasi, D. Teyssier, P. Raics, Z. L. Trocsanyi, G. Zilizi, T. Csorgo, F. Nemes, T. Novak, S. Choudhury, J. R. Komaragiri, D. Kumar, L. Panwar, P. C. Tiwari, S. Bahinipati, D. Dash, C. Kar, P. Mal, T. Mishra, V. K. Muraleedharan Nair Bindhu, A. Nayak, D. K. Sahoo, N. Sur, S. K. Swain, S. Bansal, S. B. Beri, V. Bhatnagar, S. Chauhan, N. Dhingra, R. Gupta, A. Kaur, S. Kaur, P. Kumari, M. Lohan, M. Meena, K. Sandeep, S. Sharma, J. B. Singh, A. K. Virdi, A. Ahmed, A. Bhardwaj, B. C. Choudhary, R. B. Garg, M. Gola, S. Keshri, A. Kumar, M. Naimuddin, P. Priyanka, K. Ranjan, A. Shah, M. Bharti, R. Bhattacharya, S. Bhattacharya, D. Bhowmik, S. Dutta, S. Ghosh, B. Gomber, M. Maity, S. Nandan, P. Palit, A. Purohit, P. K. Rout, G. Saha, S. Sarkar, M. Sharan, B. Singh, S. Thakur, P. K. Behera, S. C. Behera, P. Kalbhor, A. Muhammad, R. Pradhan, P. R. Pujahari, A. Sharma, A. K. Sikdar, D. Dutta, V. Kumar, K. Naskar, P. K. Netrakanti, L. M. Pant, P. Shukla, T. Aziz, M. A. Bhat, S. Dugad, R. Kumar Verma, G. B. Mohanty, U. Sarkar, S. Banerjee, S. Bhattacharya, S. Chatterjee, M. Guchait, S. Karmakar, S. Kumar, G. Majumder, K. Mazumdar, S. Mukherjee, D. Roy, N. Sahoo, S. Dube, B. Kansal, K. Kothekar, S. Pandey, A. Rane, A. Rastogi, S. Sharma, H. Bakhshiansohi, S. Chenarani, S. M. Etesami, M. Khakzad, M. Mohammadi Najafabadi, M. Felcini, M. Grunewald, M. Abbrescia, R. Aly, C. Aruta, A. Colaleo, D. Creanza, N. De Filippis, M. De Palma, A. Di Florio, A. Di Pilato, W. Elmetenawee, L. Fiore, A. Gelmi, M. Gul, G. Iaselli, M. Ince, S. Lezki, G. Maggi, M. Maggi, I. Margjeka, V. Mastrapasqua, J. A. Merlin, S. My, S. Nuzzo, A. Pompili, G. Pugliese, A. Ranieri, G. Selvaggi, L. Silvestris, F. M. Simone, R. Venditti, P. Verwilligen, G. Abbiendi, C. Battilana, D. Bonacorsi, L. Borgonovi, S. Braibant-Giacomelli, R. Campanini, P. Capiluppi, A. Castro, F. R. Cavallo, C. Ciocca, M. Cuffiani, G. M. Dallavalle, T. Diotalevi, F. Fabbri, A. Fanfani, E. Fontanesi, P. Giacomelli, L. Giommi, C. Grandi, L. Guiducci, F. Iemmi, S. Lo Meo, S. Marcellini, G. Masetti, F. L. Navarria, A. Perrotta, F. Primavera, T. Rovelli, G. P. Siroli, N. Tosi, S. Albergo, S. Costa, A. Di Mattia, R. Potenza, A. Tricomi, C. Tuve, G. Barbagli, A. Cassese, R. Ceccarelli, V. Ciulli, C. Civinini, R. D’Alessandro, F. Fiori, E. Focardi, G. Latino, P. Lenzi, M. Lizzo, M. Meschini, S. Paoletti, R. Seidita, G. Sguazzoni, L. Viliani, L. Benussi, S. Bianco, D. Piccolo, M. Bozzo, F. Ferro, R. Mulargia, E. Robutti, S. Tosi, A. Benaglia, A. Beschi, F. Brivio, F. Cetorelli, V. Ciriolo, F. De Guio, M. E. Dinardo, P. Dini, S. Gennai, A. Ghezzi, P. Govoni, L. Guzzi, M. Malberti, S. Malvezzi, D. Menasce, F. Monti, L. Moroni, M. Paganoni, D. Pedrini, S. Ragazzi, T. Tabarelli de Fatis, D. Valsecchi, D. Zuolo, S. Buontempo, N. Cavallo, A. De Iorio, F. Fabozzi, F. Fienga, A. O. M. Iorio, L. Lista, S. Meola, P. Paolucci, B. Rossi, C. Sciacca, E. Voevodina, P. Azzi, N. Bacchetta, D. Bisello, A. Boletti, A. Bragagnolo, R. Carlin, P. Checchia, P. De Castro Manzano, T. Dorigo, F. Gasparini, U. Gasparini, S. Y. Hoh, L. Layer, M. Margoni, A. T. Meneguzzo, M. Presilla, P. Ronchese, R. Rossin, F. Simonetto, G. Strong, A. Tiko, M. Tosi, H. YARAR, M. Zanetti, P. Zotto, A. Zucchetta, G. Zumerle, C. Aime‘, A. Braghieri, S. Calzaferri, D. Fiorina, P. Montagna, S. P. Ratti, V. Re, M. Ressegotti, C. Riccardi, P. Salvini, I. Vai, P. Vitulo, M. Biasini, G. M. Bilei, D. Ciangottini, L. Fanò, P. Lariccia, G. Mantovani, V. Mariani, M. Menichelli, F. Moscatelli, A. Piccinelli, A. Rossi, A. Santocchia, D. Spiga, T. Tedeschi, K. Androsov, P. Azzurri, G. Bagliesi, V. Bertacchi, L. Bianchini, T. Boccali, R. Castaldi, M. A. Ciocci, R. Dell’Orso, M. R. Di Domenico, S. Donato, L. Giannini, A. Giassi, M. T. Grippo, F. Ligabue, E. Manca, G. Mandorli, A. Messineo, F. Palla, G. Ramirez-Sanchez, A. Rizzi, G. Rolandi, S. Roy Chowdhury, A. Scribano, N. Shafiei, P. Spagnolo, R. Tenchini, G. Tonelli, N. Turini, A. Venturi, P. G. Verdini, F. Cavallari, M. Cipriani, D. Del Re, E. Di Marco, M. Diemoz, E. Longo, P. Meridiani, G. Organtini, F. Pandolfi, R. Paramatti, C. Quaranta, S. Rahatlou, C. Rovelli, F. Santanastasio, L. Soffi, R. Tramontano, N. Amapane, R. Arcidiacono, S. Argiro, M. Arneodo, N. Bartosik, R. Bellan, A. Bellora, C. Biino, A. Cappati, N. Cartiglia, S. Cometti, M. Costa, R. Covarelli, N. Demaria, B. Kiani, F. Legger, C. Mariotti, S. Maselli, E. Migliore, V. Monaco, E. Monteil, M. Monteno, M. M. Obertino, G. Ortona, L. Pacher, N. Pastrone, M. Pelliccioni, G. L. Pinna Angioni, M. Ruspa, R. Salvatico, F. Siviero, V. Sola, A. Solano, D. Soldi, A. Staiano, D. Trocino, S. Belforte, V. Candelise, M. Casarsa, F. Cossutti, A. Da Rold, G. Della Ricca, F. Vazzoler, S. Dogra, C. Huh, B. Kim, D. H. Kim, G. N. Kim, J. Lee, S. W. Lee, C. S. Moon, Y. D. Oh, S. I. Pak, B. C. Radburn-Smith, S. Sekmen, Y. C. Yang, H. Kim, D. H. Moon, B. Francois, T. J. Kim, J. Park, S. Cho, S. Choi, Y. Go, S. Ha, B. Hong, K. Lee, K. S. Lee, J. Lim, J. Park, S. K. Park, J. Yoo, J. Goh, A. Gurtu, H. S. Kim, Y. Kim, J. Almond, J. H. Bhyun, J. Choi, S. Jeon, J. Kim, J. S. Kim, S. Ko, H. Kwon, H. Lee, K. Lee, S. Lee, K. Nam, B. H. Oh, M. Oh, S. B. Oh, H. Seo, U. K. Yang, I. Yoon, D. Jeon, J. H. Kim, B. Ko, J. S. H. Lee, I. C. Park, Y. Roh, D. Song, I. J. Watson, H. D. Yoo, Y. Choi, C. Hwang, Y. Jeong, H. Lee, Y. Lee, I. Yu, Y. Maghrbi, V. Veckalns, A. Juodagalvis, A. Rinkevicius, G. Tamulaitis, W. A. T. Wan Abdullah, M. N. Yusli, Z. Zolkapli, J. F. Benitez, A. Castaneda Hernandez, J. A. Murillo Quijada, L. Valencia Palomo, G. Ayala, H. Castilla-Valdez, E. De La Cruz-Burelo, I. Heredia-De La Cruz, R. Lopez-Fernandez, D. A. Perez Navarro, A. Sanchez-Hernandez, S. Carrillo Moreno, C. Oropeza Barrera, M. Ramirez-Garcia, F. Vazquez Valencia, J. Eysermans, I. Pedraza, H. A. Salazar Ibarguen, C. Uribe Estrada, A. Morelos Pineda, J. Mijuskovic, N. Raicevic, D. Krofcheck, S. Bheesette, P. H. Butler, A. Ahmad, M. I. Asghar, M. I. M. Awan, H. R. Hoorani, W. A. Khan, M. A. Shah, M. Shoaib, M. Waqas, V. Avati, L. Grzanka, M. Malawski, H. Bialkowska, M. Bluj, B. Boimska, T. Frueboes, M. Górski, M. Kazana, M. Szleper, P. Traczyk, P. Zalewski, K. Bunkowski, A. Byszuk, K. Doroba, A. Kalinowski, M. Konecki, J. Krolikowski, M. Olszewski, M. Walczak, M. Araujo, P. Bargassa, D. Bastos, P. Faccioli, M. Gallinaro, J. Hollar, N. Leonardo, T. Niknejad, J. Seixas, K. Shchelina, O. Toldaiev, J. Varela, S. Afanasiev, P. Bunin, M. Gavrilenko, I. Golutvin, I. Gorbunov, A. Kamenev, V. Karjavine, A. Lanev, A. Malakhov, V. Matveev, P. Moisenz, V. Palichik, V. Perelygin, M. Savina, D. Seitova, V. Shalaev, S. Shmatov, S. Shulha, V. Smirnov, O. Teryaev, N. Voytishin, A. Zarubin, I. Zhizhin, G. Gavrilov, V. Golovtcov, Y. Ivanov, V. Kim, E. Kuznetsova, V. Murzin, V. Oreshkin, I. Smirnov, D. Sosnov, V. Sulimov, L. Uvarov, S. Volkov, A. Vorobyev, Yu. Andreev, A. Dermenev, S. Gninenko, N. Golubev, A. Karneyeu, M. Kirsanov, N. Krasnikov, A. Pashenkov, G. Pivovarov, D. Tlisov, A. Toropin, V. Epshteyn, V. Gavrilov, N. Lychkovskaya, A. Nikitenko, V. Popov, G. Safronov, A. Spiridonov, A. Stepennov, M. Toms, E. Vlasov, A. Zhokin, T. Aushev, O. Bychkova, M. Chadeeva, R. Chistov, P. Parygin, E. Popova, V. Andreev, M. Azarkin, I. Dremin, M. Kirakosyan, A. Terkulov, A. Belyaev, E. Boos, V. Bunichev, M. Dubinin, L. Dudko, A. Ershov, V. Klyukhin, O. Kodolova, I. Lokhtin, S. Obraztsov, S. Petrushanko, V. Savrin, A. Snigirev, V. Blinov, T. Dimova, L. Kardapoltsev, I. Ovtin, Y. Skovpen, I. Azhgirey, I. Bayshev, V. Kachanov, A. Kalinin, D. Konstantinov, V. Petrov, R. Ryutin, A. Sobol, S. Troshin, N. Tyurin, A. Uzunian, A. Volkov, A. Babaev, A. Iuzhakov, V. Okhotnikov, L. Sukhikh, V. Borchsh, V. Ivanchenko, E. Tcherniaev, P. Adzic, P. Cirkovic, M. Dordevic, P. Milenovic, J. Milosevic, M. Aguilar-Benitez, J. Alcaraz Maestre, A. Álvarez Fernández, I. Bachiller, M. Barrio Luna, Cristina F. Bedoya, J. A. Brochero Cifuentes, C. A. Carrillo Montoya, M. Cepeda, M. Cerrada, N. Colino, B. De La Cruz, A. Delgado Peris, J. P. Fernández Ramos, J. Flix, M. C. Fouz, A. García Alonso, O. Gonzalez Lopez, S. Goy Lopez, J. M. Hernandez, M. I. Josa, J. León Holgado, D. Moran, Á. Navarro Tobar, A. Pérez-Calero Yzquierdo, J. Puerta Pelayo, I. Redondo, L. Romero, S. Sánchez Navas, M. S. Soares, A. Triossi, L. Urda Gómez, C. Willmott, C. Albajar, J. F. de Trocóniz, R. Reyes-Almanza, B. Alvarez Gonzalez, J. Cuevas, C. Erice, J. Fernandez Menendez, S. Folgueras, I. Gonzalez Caballero, E. Palencia Cortezon, C. Ramón Álvarez, J. Ripoll Sau, V. Rodríguez Bouza, S. Sanchez Cruz, A. Trapote, I. J. Cabrillo, A. Calderon, B. Chazin Quero, J. Duarte Campderros, M. Fernandez, P. J. Fernández Manteca, G. Gomez, C. Martinez Rivero, P. Martinez Ruiz del Arbol, F. Matorras, J. Piedra Gomez, C. Prieels, F. Ricci-Tam, T. Rodrigo, A. Ruiz-Jimeno, L. Scodellaro, I. Vila, J. M. Vizan Garcia, MK Jayananda, B. Kailasapathy, D. U. J. Sonnadara, DDC Wickramarathna, W. G. D. Dharmaratna, K. Liyanage, N. Perera, N. Wickramage, T. K. Aarrestad, D. Abbaneo, B. Akgun, E. Auffray, G. Auzinger, J. Baechler, P. Baillon, A. H. Ball, D. Barney, J. Bendavid, N. Beni, M. Bianco, A. Bocci, P. Bortignon, E. Bossini, E. Brondolin, T. Camporesi, G. Cerminara, L. Cristella, D. d’Enterria, A. Dabrowski, N. Daci, V. Daponte, A. David, A. De Roeck, M. Deile, R. Di Maria, M. Dobson, M. Dünser, N. Dupont, A. Elliott-Peisert, N. Emriskova, F. Fallavollita, D. Fasanella, S. Fiorendi, A. Florent, G. Franzoni, J. Fulcher, W. Funk, S. Giani, D. Gigi, K. Gill, F. Glege, L. Gouskos, M. Guilbaud, D. Gulhan, M. Haranko, J. Hegeman, Y. Iiyama, V. Innocente, T. James, P. Janot, J. Kaspar, J. Kieseler, M. Komm, N. Kratochwil, C. Lange, P. Lecoq, K. Long, C. Lourenço, L. Malgeri, M. Mannelli, A. Massironi, F. Meijers, S. Mersi, E. Meschi, F. Moortgat, M. Mulders, J. Ngadiuba, J. Niedziela, S. Orfanelli, L. Orsini, F. Pantaleo, L. Pape, E. Perez, M. Peruzzi, A. Petrilli, G. Petrucciani, A. Pfeiffer, M. Pierini, D. Rabady, A. Racz, M. Rieger, M. Rovere, H. Sakulin, J. Salfeld-Nebgen, S. Scarfi, C. Schäfer, C. Schwick, M. Selvaggi, A. Sharma, P. Silva, W. Snoeys, P. Sphicas, J. Steggemann, S. Summers, V. R. Tavolaro, D. Treille, A. Tsirou, G. P. Van Onsem, A. Vartak, M. Verzetti, K. A. Wozniak, W. D. Zeuner, L. Caminada, W. Erdmann, R. Horisberger, Q. Ingram, H. C. Kaestli, D. Kotlinski, U. Langenegger, T. Rohe, M. Backhaus, P. Berger, A. Calandri, N. Chernyavskaya, A. De Cosa, G. Dissertori, M. Dittmar, M. Donegà, C. Dorfer, T. Gadek, T. A. Gómez Espinosa, C. Grab, D. Hits, W. Lustermann, A.-M. Lyon, R. A. Manzoni, M. T. Meinhard, F. Micheli, F. Nessi-Tedaldi, F. Pauss, V. Perovic, G. Perrin, L. Perrozzi, S. Pigazzini, M. G. Ratti, M. Reichmann, C. Reissel, T. Reitenspiess, B. Ristic, D. Ruini, D. A. Sanz Becerra, M. Schönenberger, V. Stampf, M. L. Vesterbacka Olsson, R. Wallny, D. H. Zhu, C. Amsler, C. Botta, D. Brzhechko, M. F. Canelli, R. Del Burgo, J. K. Heikkilä, M. Huwiler, A. Jofrehei, B. Kilminster, S. Leontsinis, A. Macchiolo, P. Meiring, V. M. Mikuni, U. Molinatti, I. Neutelings, G. Rauco, A. Reimers, P. Robmann, K. Schweiger, Y. Takahashi, S. Wertz, C. Adloff, C. M. Kuo, W. Lin, A. Roy, T. Sarkar, S. S. Yu, L. Ceard, P. Chang, Y. Chao, K. F. Chen, P. H. Chen, W.-S. Hou, Y. y. Li, R.-S. Lu, E. Paganis, A. Psallidas, A. Steen, E. Yazgan, B. Asavapibhop, C. Asawatangtrakuldee, N. Srimanobhas, F. Boran, S. Damarseckin, Z. S. Demiroglu, F. Dolek, C. Dozen, I. Dumanoglu, E. Eskut, G. Gokbulut, Y. Guler, E. Gurpinar Guler, I. Hos, C. Isik, E. E. Kangal, O. Kara, A. Kayis Topaksu, U. Kiminsu, G. Onengut, K. Ozdemir, A. Polatoz, A. E. Simsek, B. Tali, U. G. Tok, S. Turkcapar, I. S. Zorbakir, C. Zorbilmez, B. Isildak, G. Karapinar, K. Ocalan, M. Yalvac, I. O. Atakisi, E. Gülmez, M. Kaya, O. Kaya, Ö. Özçelik, S. Tekten, E. A. Yetkin, A. Cakir, K. Cankocak, Y. Komurcu, S. Sen, F. Aydogmus Sen, S. Cerci, B. Kaynak, S. Ozkorucuklu, D. Sunar Cerci, B. Grynyov, L. Levchuk, E. Bhal, S. Bologna, J. J. Brooke, E. Clement, D. Cussans, H. Flacher, J. Goldstein, G. P. Heath, H. F. Heath, L. Kreczko, B. Krikler, S. Paramesvaran, T. Sakuma, S. Seif El Nasr-Storey, V. J. Smith, J. Taylor, A. Titterton, K. W. Bell, A. Belyaev, C. Brew, R. M. Brown, D. J. A. Cockerill, K. V. Ellis, K. Harder, S. Harper, J. Linacre, K. Manolopoulos, D. M. Newbold, E. Olaiya, D. Petyt, T. Reis, T. Schuh, C. H. Shepherd-Themistocleous, A. Thea, I. R. Tomalin, T. Williams, R. Bainbridge, P. Bloch, S. Bonomally, J. Borg, S. Breeze, O. Buchmuller, A. Bundock, V. Cepaitis, G. S. Chahal, D. Colling, P. Dauncey, G. Davies, M. Della Negra, G. Fedi, G. Hall, G. Iles, J. Langford, L. Lyons, A.-M. Magnan, S. Malik, A. Martelli, V. Milosevic, J. Nash, V. Palladino, M. Pesaresi, D. M. Raymond, A. Richards, A. Rose, E. Scott, C. Seez, A. Shtipliyski, M. Stoye, A. Tapper, K. Uchida, T. Virdee, N. Wardle, S. N. Webb, D. Winterbottom, A. G. Zecchinelli, J. E. Cole, P. R. Hobson, A. Khan, P. Kyberd, C. K. Mackay, I. D. Reid, L. Teodorescu, S. Zahid, A. Brinkerhoff, K. Call, B. Caraway, J. Dittmann, K. Hatakeyama, A. R. Kanuganti, C. Madrid, B. McMaster, N. Pastika, S. Sawant, C. Smith, J. Wilson, R. Bartek, A. Dominguez, R. Uniyal, A. M. Vargas Hernandez, A. Buccilli, O. Charaf, S. I. Cooper, S. V. Gleyzer, C. Henderson, P. Rumerio, C. West, A. Akpinar, A. Albert, D. Arcaro, C. Cosby, Z. Demiragli, D. Gastler, C. Richardson, J. Rohlf, K. Salyer, D. Sperka, D. Spitzbart, I. Suarez, S. Yuan, D. Zou, G. Benelli, B. Burkle, X. Coubez, D. Cutts, Y. t. Duh, M. Hadley, U. Heintz, J. M. Hogan, K. H. M. Kwok, E. Laird, G. Landsberg, K. T. Lau, J. Lee, M. Narain, S. Sagir, R. Syarif, E. Usai, W. Y. Wong, D. Yu, W. Zhang, R. Band, C. Brainerd, R. Breedon, M. Calderon De La Barca Sanchez, M. Chertok, J. Conway, R. Conway, P. T. Cox, R. Erbacher, C. Flores, G. Funk, F. Jensen, W. Ko, O. Kukral, R. Lander, M. Mulhearn, D. Pellett, J. Pilot, M. Shi, D. Taylor, K. Tos, M. Tripathi, Y. Yao, F. Zhang, M. Bachtis, R. Cousins, A. Dasgupta, D. Hamilton, J. Hauser, M. Ignatenko, T. Lam, N. Mccoll, W. A. Nash, S. Regnard, D. Saltzberg, C. Schnaible, B. Stone, V. Valuev, K. Burt, Y. Chen, R. Clare, J. W. Gary, S. M. A. Ghiasi Shirazi, G. Hanson, G. Karapostoli, O. R. Long, N. Manganelli, M. Olmedo Negrete, M. I. Paneva, W. Si, S. Wimpenny, Y. Zhang, J. G. Branson, P. Chang, S. Cittolin, S. Cooperstein, N. Deelen, M. Derdzinski, J. Duarte, R. Gerosa, D. Gilbert, B. Hashemi, V. Krutelyov, J. Letts, M. Masciovecchio, S. May, S. Padhi, M. Pieri, V. Sharma, M. Tadel, F. Würthwein, A. Yagil, N. Amin, C. Campagnari, M. Citron, A. Dorsett, V. Dutta, J. Incandela, B. Marsh, H. Mei, A. Ovcharova, H. Qu, M. Quinnan, J. Richman, U. Sarica, D. Stuart, S. Wang, D. Anderson, A. Bornheim, O. Cerri, I. Dutta, J. M. Lawhorn, N. Lu, J. Mao, H. B. Newman, T. Q. Nguyen, J. Pata, M. Spiropulu, J. R. Vlimant, S. Xie, Z. Zhang, R. Y. Zhu, J. Alison, M. B. Andrews, T. Ferguson, T. Mudholkar, M. Paulini, M. Sun, I. Vorobiev, J. P. Cumalat, W. T. Ford, E. MacDonald, T. Mulholland, R. Patel, A. Perloff, K. Stenson, K. A. Ulmer, S. R. Wagner, J. Alexander, Y. Cheng, J. Chu, D. J. Cranshaw, A. Datta, A. Frankenthal, K. Mcdermott, J. Monroy, J. R. Patterson, D. Quach, A. Ryd, W. Sun, S. M. Tan, Z. Tao, J. Thom, P. Wittich, M. Zientek, S. Abdullin, M. Albrow, M. Alyari, G. Apollinari, A. Apresyan, A. Apyan, S. Banerjee, L. A. T. Bauerdick, A. Beretvas, D. Berry, J. Berryhill, P. C. Bhat, K. Burkett, J. N. Butler, A. Canepa, G. B. Cerati, H. W. K. Cheung, F. Chlebana, M. Cremonesi, V. D. Elvira, J. Freeman, Z. Gecse, E. Gottschalk, L. Gray, D. Green, S. Grünendahl, O. Gutsche, R. M. Harris, S. Hasegawa, R. Heller, T. C. Herwig, J. Hirschauer, B. Jayatilaka, S. Jindariani, M. Johnson, U. Joshi, P. Klabbers, T. Klijnsma, B. Klima, M. J. Kortelainen, S. Lammel, D. Lincoln, R. Lipton, M. Liu, T. Liu, J. Lykken, K. Maeshima, D. Mason, P. McBride, P. Merkel, S. Mrenna, S. Nahn, V. O’Dell, V. Papadimitriou, K. Pedro, C. Pena, O. Prokofyev, F. Ravera, A. Reinsvold Hall, L. Ristori, B. Schneider, E. Sexton-Kennedy, N. Smith, A. Soha, W. J. Spalding, L. Spiegel, S. Stoynev, J. Strait, L. Taylor, S. Tkaczyk, N. V. Tran, L. Uplegger, E. W. Vaandering, H. A. Weber, A. Woodard, D. Acosta, P. Avery, D. Bourilkov, L. Cadamuro, V. Cherepanov, F. Errico, R. D. Field, D. Guerrero, B. M. Joshi, M. Kim, J. Konigsberg, A. Korytov, K. H. Lo, K. Matchev, N. Menendez, G. Mitselmakher, D. Rosenzweig, K. Shi, J. Wang, S. Wang, X. Zuo, T. Adams, A. Askew, D. Diaz, R. Habibullah, S. Hagopian, V. Hagopian, K. F. Johnson, R. Khurana, T. Kolberg, G. Martinez, H. Prosper, C. Schiber, R. Yohay, J. Zhang, M. M. Baarmand, S. Butalla, T. Elkafrawy, M. Hohlmann, D. Noonan, M. Rahmani, M. Saunders, F. Yumiceva, M. R. Adams, L. Apanasevich, H. Becerril Gonzalez, R. Cavanaugh, X. Chen, S. Dittmer, O. Evdokimov, C. E. Gerber, D. A. Hangal, D. J. Hofman, C. Mills, G. Oh, T. Roy, M. B. Tonjes, N. Varelas, J. Viinikainen, X. Wang, Z. Wu, M. Alhusseini, K. Dilsiz, S. Durgut, R. P. Gandrajula, M. Haytmyradov, V. Khristenko, O. K. Köseyan, J.-P. Merlo, A. Mestvirishvili, A. Moeller, J. Nachtman, H. Ogul, Y. Onel, F. Ozok, A. Penzo, C. Snyder, E. Tiras, J. Wetzel, K. Yi, O. Amram, B. Blumenfeld, L. Corcodilos, M. Eminizer, A. V. Gritsan, S. Kyriacou, P. Maksimovic, C. Mantilla, J. Roskes, M. Swartz, T.Á. Vámi, C. Baldenegro Barrera, P. Baringer, A. Bean, A. Bylinkin, T. Isidori, S. Khalil, J. King, G. Krintiras, A. Kropivnitskaya, C. Lindsey, N. Minafra, M. Murray, C. Rogan, C. Royon, S. Sanders, E. Schmitz, J. D. Tapia Takaki, Q. Wang, J. Williams, G. Wilson, S. Duric, A. Ivanov, K. Kaadze, D. Kim, Y. Maravin, T. Mitchell, A. Modak, A. Mohammadi, F. Rebassoo, D. Wright, E. Adams, A. Baden, O. Baron, A. Belloni, S. C. Eno, Y. Feng, N. J. Hadley, S. Jabeen, G. Y. Jeng, R. G. Kellogg, T. Koeth, A. C. Mignerey, S. Nabili, M. Seidel, A. Skuja, S. C. Tonwar, L. Wang, K. Wong, D. Abercrombie, B. Allen, R. Bi, S. Brandt, W. Busza, I. A. Cali, Y. Chen, M. D’Alfonso, G. Gomez Ceballos, M. Goncharov, P. Harris, D. Hsu, M. Hu, M. Klute, D. Kovalskyi, J. Krupa, Y.-J. Lee, P. D. Luckey, B. Maier, A. C. Marini, C. Mcginn, C. Mironov, S. Narayanan, X. Niu, C. Paus, D. Rankin, C. Roland, G. Roland, Z. Shi, G. S. F. Stephans, K. Sumorok, K. Tatar, D. Velicanu, J. Wang, T. W. Wang, Z. Wang, B. Wyslouch, R. M. Chatterjee, A. Evans, S. Guts, P. Hansen, J. Hiltbrand, Sh. Jain, M. Krohn, Y. Kubota, Z. Lesko, J. Mans, M. Revering, R. Rusack, R. Saradhy, N. Schroeder, N. Strobbe, M. A. Wadud, J. G. Acosta, S. Oliveros, K. Bloom, S. Chauhan, D. R. Claes, C. Fangmeier, L. Finco, F. Golf, J. R. González Fernández, I. Kravchenko, J. E. Siado, G. R. Snow, B. Stieger, W. Tabb, F. Yan, G. Agarwal, H. Bandyopadhyay, C. Harrington, L. Hay, I. Iashvili, A. Kharchilava, C. McLean, D. Nguyen, J. Pekkanen, S. Rappoccio, B. Roozbahani, G. Alverson, E. Barberis, C. Freer, Y. Haddad, A. Hortiangtham, J. Li, G. Madigan, B. Marzocchi, D. M. Morse, V. Nguyen, T. Orimoto, A. Parker, L. Skinnari, A. Tishelman-Charny, T. Wamorkar, B. Wang, A. Wisecarver, D. Wood, S. Bhattacharya, J. Bueghly, Z. Chen, A. Gilbert, T. Gunter, K. A. Hahn, N. Odell, M. H. Schmitt, K. Sung, M. Velasco, R. Bucci, N. Dev, R. Goldouzian, M. Hildreth, K. Hurtado Anampa, C. Jessop, D. J. Karmgard, K. Lannon, W. Li, N. Loukas, N. Marinelli, I. Mcalister, F. Meng, K. Mohrman, Y. Musienko, R. Ruchti, P. Siddireddy, S. Taroni, M. Wayne, A. Wightman, M. Wolf, L. Zygala, J. Alimena, B. Bylsma, B. Cardwell, L. S. Durkin, B. Francis, C. Hill, A. Lefeld, B. L. Winer, B. R. Yates, P. Das, G. Dezoort, P. Elmer, B. Greenberg, N. Haubrich, S. Higginbotham, A. Kalogeropoulos, G. Kopp, S. Kwan, D. Lange, M. T. Lucchini, J. Luo, D. Marlow, K. Mei, I. Ojalvo, J. Olsen, C. Palmer, P. Piroué, D. Stickland, C. Tully, S. Malik, S. Norberg, V. E. Barnes, R. Chawla, S. Das, L. Gutay, M. Jones, A. W. Jung, B. Mahakud, G. Negro, N. Neumeister, C. C. Peng, S. Piperov, H. Qiu, J. F. Schulte, M. Stojanovic, N. Trevisani, F. Wang, R. Xiao, W. Xie, T. Cheng, J. Dolen, N. Parashar, A. Baty, S. Dildick, K. M. Ecklund, S. Freed, F. J. M. Geurts, M. Kilpatrick, A. Kumar, W. Li, B. P. Padley, R. Redjimi, J. Roberts, J. Rorie, W. Shi, A. G. Stahl Leiton, A. Bodek, P. de Barbaro, R. Demina, J. L. Dulemba, C. Fallon, T. Ferbel, M. Galanti, A. Garcia-Bellido, O. Hindrichs, A. Khukhunaishvili, E. Ranken, R. Taus, B. Chiarito, J. P. Chou, A. Gandrakota, Y. Gershtein, E. Halkiadakis, A. Hart, M. Heindl, E. Hughes, S. Kaplan, O. Karacheban, I. Laflotte, A. Lath, R. Montalvo, K. Nash, M. Osherson, S. Salur, S. Schnetzer, S. Somalwar, R. Stone, S. A. Thayil, S. Thomas, H. Wang, H. Acharya, A. G. Delannoy, S. Spanier, O. Bouhali, M. Dalchenko, A. Delgado, R. Eusebi, J. Gilmore, T. Huang, T. Kamon, H. Kim, S. Luo, S. Malhotra, R. Mueller, D. Overton, L. Perniè, D. Rathjens, A. Safonov, J. Sturdy, N. Akchurin, J. Damgov, V. Hegde, S. Kunori, K. Lamichhane, S. W. Lee, T. Mengke, S. Muthumuni, T. Peltola, S. Undleeb, I. Volobouev, Z. Wang, A. Whitbeck, E. Appelt, S. Greene, A. Gurrola, R. Janjam, W. Johns, C. Maguire, A. Melo, H. Ni, K. Padeken, F. Romeo, P. Sheldon, S. Tuo, J. Velkovska, M. Verweij, M. W. Arenton, B. Cox, G. Cummings, J. Hakala, R. Hirosky, M. Joyce, A. Ledovskoy, A. Li, C. Neu, B. Tannenwald, Y. Wang, E. Wolfe, F. Xia, R. Harr, P. E. Karchin, N. Poudyal, P. Thapa, K. Black, T. Bose, J. Buchanan, C. Caillol, S. Dasu, I. De Bruyn, P. Everaerts, C. Galloni, H. He, M. Herndon, A. Hervé, U. Hussain, A. Lanaro, A. Loeliger, R. Loveless, J. Madhusudanan Sreekala, A. Mallampalli, D. Pinna, T. Ruggles, A. Savin, V. Shang, V. Sharma, W. H. Smith, D. Teague, S. Trembath-reichert, W. Vetens, CMS Collaboration

**Affiliations:** 1 Yerevan Physics Institute, Yerevan, Armenia; 2 Institut für Hochenergiephysik, Wien, Austria; 3 Institute for Nuclear Problems, Minsk, Belarus; 4 Universiteit Antwerpen, Antwerpen, Belgium; 5 Vrije Universiteit Brussel, Brussel, Belgium; 6 Université Libre de Bruxelles, Brussels, Belgium; 7 Ghent University, Ghent, Belgium; 8 Université Catholique de Louvain, Louvain-la-Neuve, Belgium; 9 Centro Brasileiro de Pesquisas Fisicas, Rio de Janeiro, Brazil; 10 Universidade do Estado do Rio de Janeiro, Rio de Janeiro, Brazil; 11 Universidade Estadual Paulista, Universidade Federal do ABC, São Paulo, Brazil; 12 Institute for Nuclear Research and Nuclear Energy, Bulgarian Academy of Sciences, Sofia, Bulgaria; 13 University of Sofia, Sofia, Bulgaria; 14 Beihang University, Beijing, China; 15 Department of Physics, Tsinghua University, Beijing, China; 16 Institute of High Energy Physics, Beijing, China; 17 State Key Laboratory of Nuclear Physics and Technology, Peking University, Beijing, China; 18 Sun Yat-Sen University, Guangzhou, China; 19 Institute of Modern Physics and Key Laboratory of Nuclear Physics and Ion-beam Application (MOE)-Fudan University, Shanghai, China; 20 Zhejiang University, Hangzhou, China; 21 Universidad de Los Andes, Bogota, Colombia; 22 Universidad de Antioquia, Medellin, Colombia; 23 Faculty of Electrical Engineering, Mechanical Engineering and Naval Architecture, University of Split, Split, Croatia; 24 University of Split, Faculty of Science, Split, Croatia; 25 Institute Rudjer Boskovic, Zagreb, Croatia; 26 University of Cyprus, Nicosia, Cyprus; 27 Charles University, Prague, Czech Republic; 28 Escuela Politecnica Nacional, Quito, Ecuador; 29 Universidad San Francisco de Quito, Quito, Ecuador; 30 Academy of Scientific Research and Technology of the Arab Republic of Egypt, Egyptian Network of High Energy Physics, Cairo, Egypt; 31 Center for High Energy Physics (CHEP-FU), Fayoum University, El-Fayoum, Egypt; 32 National Institute of Chemical Physics and Biophysics, Tallinn, Estonia; 33 Department of Physics, University of Helsinki, Helsinki, Finland; 34 Helsinki Institute of Physics, Helsinki, Finland; 35 Lappeenranta University of Technology, Lappeenranta, Finland; 36 IRFU, CEA, Université Paris-Saclay, Gif-sur-Yvette, France; 37 Laboratoire Leprince-Ringuet, CNRS/IN2P3, Ecole Polytechnique, Institut Polytechnique de Paris, Palaiseau, France; 38 Université de Strasbourg, CNRS, IPHC UMR 7178, Strasbourg, France; 39 Université de Lyon, Université Claude Bernard Lyon 1, CNRS-IN2P3, Institut de Physique Nucléaire de Lyon, Villeurbanne, France; 40 Georgian Technical University, Tbilisi, Georgia; 41 I. Physikalisches Institut, RWTH Aachen University, Aachen, Germany; 42 III. Physikalisches Institut A, RWTH Aachen University, Aachen, Germany; 43 III. Physikalisches Institut B, RWTH Aachen University, Aachen, Germany; 44 Deutsches Elektronen-Synchrotron, Hamburg, Germany; 45 University of Hamburg, Hamburg, Germany; 46 Karlsruher Institut fuer Technologie, Karlsruhe, Germany; 47 Institute of Nuclear and Particle Physics (INPP), NCSR Demokritos, Aghia Paraskevi, Greece; 48 National and Kapodistrian University of Athens, Athens, Greece; 49 National Technical University of Athens, Athens, Greece; 50 University of Ioánnina, Ioannina, Greece; 51 MTA-ELTE Lendület CMS Particle and Nuclear Physics Group, Eötvös Loránd University, Budapest, Hungary; 52 Wigner Research Centre for Physics, Budapest, Hungary; 53 Institute of Nuclear Research ATOMKI, Debrecen, Hungary; 54 Institute of Physics, University of Debrecen, Debrecen, Hungary; 55 Eszterhazy Karoly University, Karoly Robert Campus, Gyongyos, Hungary; 56 Indian Institute of Science (IISc), Bangalore, India; 57 National Institute of Science Education and Research, HBNI, Bhubaneswar, India; 58 Panjab University, Chandigarh, India; 59 University of Delhi, Delhi, India; 60 Saha Institute of Nuclear Physics, HBNI, Kolkata, India; 61 Indian Institute of Technology Madras, Madras, India; 62 Bhabha Atomic Research Centre, Mumbai, India; 63 Tata Institute of Fundamental Research-A, Mumbai, India; 64 Tata Institute of Fundamental Research-B, Mumbai, India; 65 Indian Institute of Science Education and Research (IISER), Pune, India; 66 Department of Physics, Isfahan University of Technology, Isfahan, Iran; 67 Institute for Research in Fundamental Sciences (IPM), Tehran, Iran; 68 University College Dublin, Dublin, Ireland; 69 INFN Sezione di Bari , Università di Bari, Politecnico di Bari, Bari, Italy; 70 INFN Sezione di Bologna, Università di Bologna, Bologna, Italy; 71 INFN Sezione di Catania, Università di Catania, Catania, Italy; 72 INFN Sezione di Firenze, Università di Firenze, Firenze, Italy; 73 INFN Laboratori Nazionali di Frascati, Frascati, Italy; 74 INFN Sezione di Genova, Università di Genova, Genoa, Italy; 75 INFN Sezione di Milano-Bicocca, Università di Milano-Bicocca, Milan, Italy; 76 INFN Sezione di Napoli , Università di Napoli ’Federico II’ , Napoli, Italy, Università della Basilicata , Potenza, Italy, Università G. Marconi, Rome, Italy; 77 INFN Sezione di Padova , Università di Padova , Padova, Italy, Università di Trento, Trento, Italy; 78 INFN Sezione di Pavia, Università di Pavia, Pavia, Italy; 79 INFN Sezione di Perugia, Università di Perugia, Perugia, Italy; 80 INFN Sezione di Pisa , Università di Pisa, Scuola Normale Superiore di Pisa, Pisa, Italy; 81 INFN Sezione di Roma, Sapienza Università di Roma, Rome, Italy; 82 INFN Sezione di Torino , Università di Torino , Torino, Italy, Università del Piemonte Orientale, Novara, Italy; 83 INFN Sezione di Trieste, Università di Trieste, Trieste, Italy; 84 Kyungpook National University, Daegu, Korea; 85 Chonnam National University, Institute for Universe and Elementary Particles, Kwangju, Korea; 86 Hanyang University, Seoul, Korea; 87 Korea University, Seoul, Korea; 88 Department of Physics, Kyung Hee University, Seoul, Republic of Korea; 89 Sejong University, Seoul, Korea; 90 Seoul National University, Seoul, Korea; 91 University of Seoul, Seoul, Korea; 92 Department of Physics, Yonsei University, Seoul, Korea; 93 Sungkyunkwan University, Suwon, Korea; 94 College of Engineering and Technology, American University of the Middle East (AUM), Egaila, Kuwait; 95 Riga Technical University, Riga, Latvia; 96 Vilnius University, Vilnius, Lithuania; 97 National Centre for Particle Physics, Universiti Malaya, Kuala Lumpur, Malaysia; 98 Universidad de Sonora (UNISON), Hermosillo, Mexico; 99 Centro de Investigacion y de Estudios Avanzados del IPN, Mexico City, Mexico; 100 Universidad Iberoamericana, Mexico City, Mexico; 101 Benemerita Universidad Autonoma de Puebla, Puebla, Mexico; 102 Universidad Autónoma de San Luis Potosí, San Luis Potosí, Mexico; 103 University of Montenegro, Podgorica, Montenegro; 104 University of Auckland, Auckland, New Zealand; 105 University of Canterbury, Christchurch, New Zealand; 106 National Centre for Physics, Quaid-I-Azam University, Islamabad, Pakistan; 107 AGH University of Science and Technology Faculty of Computer Science, Electronics and Telecommunications, Kraków, Poland; 108 National Centre for Nuclear Research, Swierk, Poland; 109 Institute of Experimental Physics, Faculty of Physics, University of Warsaw, Warsaw, Poland; 110 Laboratório de Instrumentação e Física Experimental de Partículas, Lisbon, Portugal; 111 Joint Institute for Nuclear Research, Dubna, Russia; 112 Petersburg Nuclear Physics Institute, Gatchina (St. Petersburg), Russia; 113 Institute for Nuclear Research, Moscow, Russia; 114 Institute for Theoretical and Experimental Physics named by A.I. Alikhanov of NRC ‘Kurchatov Institute’, Moscow, Russia; 115 Moscow Institute of Physics and Technology, Moscow, Russia; 116 National Research Nuclear University ‘Moscow Engineering Physics Institute’ (MEPhI), Moscow, Russia; 117 P.N. Lebedev Physical Institute, Moscow, Russia; 118 Skobeltsyn Institute of Nuclear Physics, Lomonosov Moscow State University, Moscow, Russia; 119 Novosibirsk State University (NSU), Novosibirsk, Russia; 120 Institute for High Energy Physics of National Research Centre ‘Kurchatov Institute’, Protvino, Russia; 121 National Research Tomsk Polytechnic University, Tomsk, Russia; 122 Tomsk State University, Tomsk, Russia; 123 University of Belgrade: Faculty of Physics and VINCA Institute of Nuclear Sciences, Belgrade, Serbia; 124 Centro de Investigaciones Energéticas Medioambientales y Tecnológicas (CIEMAT), Madrid, Spain; 125 Universidad Autónoma de Madrid, Madrid, Spain; 126 Universidad de Oviedo, Instituto Universitario de Ciencias y Tecnologías Espaciales de Asturias (ICTEA), Oviedo, Spain; 127 Instituto de Física de Cantabria (IFCA), CSIC-Universidad de Cantabria, Santander, Spain; 128 University of Colombo, Colombo, Sri Lanka; 129 Department of Physics, University of Ruhuna, Matara, Sri Lanka; 130 CERN, European Organization for Nuclear Research, Geneva, Switzerland; 131 Paul Scherrer Institut, Villigen, Switzerland; 132 ETH Zurich-Institute for Particle Physics and Astrophysics (IPA), Zurich, Switzerland; 133 Universität Zürich, Zurich, Switzerland; 134 National Central University, Chung-Li, Taiwan; 135 National Taiwan University (NTU), Taipei, Taiwan; 136 Department of Physics, Faculty of Science, Chulalongkorn University, Bangkok, Thailand; 137 Physics Department, Science and Art Faculty, Çukurova University, Adana, Turkey; 138 Physics Department, Middle East Technical University, Ankara, Turkey; 139 Bogazici University, Istanbul, Turkey; 140 Istanbul Technical University, Istanbul, Turkey; 141 Istanbul University, Istanbul, Turkey; 142 Institute for Scintillation Materials of National Academy of Science of Ukraine, Kharkov, Ukraine; 143 National Scientific Center, Kharkov Institute of Physics and Technology, Kharkov, Ukraine; 144 University of Bristol, Bristol, UK; 145 Rutherford Appleton Laboratory, Didcot, UK; 146 Imperial College, London, UK; 147 Brunel University, Uxbridge, UK; 148 Baylor University, Waco, USA; 149 Catholic University of America, Washington, DC USA; 150 The University of Alabama, Tuscaloosa, USA; 151 Boston University, Boston, USA; 152 Brown University, Providence, USA; 153 University of California, Davis, Davis, USA; 154 University of California, Los Angeles, USA; 155 University of California, Riverside, Riverside, USA; 156 University of California, San Diego, La Jolla, USA; 157 Department of Physics, University of California, Santa Barbara, Santa Barbara, USA; 158 California Institute of Technology, Pasadena, USA; 159 Carnegie Mellon University, Pittsburgh, USA; 160 University of Colorado Boulder, Boulder, USA; 161 Cornell University, Ithaca, USA; 162 Fermi National Accelerator Laboratory, Batavia, USA; 163 University of Florida, Gainesville, USA; 164 Florida State University, Tallahassee, USA; 165 Florida Institute of Technology, Melbourne, USA; 166 University of Illinois at Chicago (UIC), Chicago, USA; 167 The University of Iowa, Iowa City, USA; 168 Johns Hopkins University, Baltimore, USA; 169 The University of Kansas, Lawrence, USA; 170 Kansas State University, Manhattan, USA; 171 Lawrence Livermore National Laboratory, Livermore, USA; 172 University of Maryland, College Park, USA; 173 Massachusetts Institute of Technology, Cambridge, USA; 174 University of Minnesota, Minneapolis, USA; 175 University of Mississippi, Oxford, USA; 176 University of Nebraska-Lincoln, Lincoln, USA; 177 State University of New York at Buffalo, Buffalo, USA; 178 Northeastern University, Boston, USA; 179 Northwestern University, Evanston, USA; 180 University of Notre Dame, Notre Dame, USA; 181 The Ohio State University, Columbus, USA; 182 Princeton University, Princeton, USA; 183 University of Puerto Rico, Mayaguez, USA; 184 Purdue University, West Lafayette, USA; 185 Purdue University Northwest, Hammond, USA; 186 Rice University, Houston, USA; 187 University of Rochester, Rochester, USA; 188 Rutgers, The State University of New Jersey, Piscataway, USA; 189 University of Tennessee, Knoxville, USA; 190 Texas A&M University, College Station, USA; 191 Texas Tech University, Lubbock, USA; 192 Vanderbilt University, Nashville, USA; 193 University of Virginia, Charlottesville, USA; 194 Wayne State University, Detroit, USA; 195 University of Wisconsin-Madison, Madison, WI USA; 196 Vienna University of Technology, Vienna, Austria; 197 Institute of Basic and Applied Sciences, Faculty of Engineering, Arab Academy for Science, Technology and Maritime Transport, Alexandria, Egypt; 198 Université Libre de Bruxelles, Brussels, Belgium; 199 IRFU, CEA, Université Paris-Saclay, Gif-sur-Yvette, France; 200 Universidade Estadual de Campinas, Campinas, Brazil; 201 Federal University of Rio Grande do Sul, Porto Alegre, Brazil; 202 UFMS, Nova Andradina, Brazil; 203 Universidade Federal de Pelotas, Pelotas, Brazil; 204 University of Chinese Academy of Sciences, Beijing, China; 205 Institute for Theoretical and Experimental Physics named by A.I. Alikhanov of NRC ‘Kurchatov Institute’, Moscow, Russia; 206 Joint Institute for Nuclear Research, Dubna, Russia; 207 Cairo University, Cairo, Egypt; 208 Suez University, Suez, Egypt; 209 British University in Egypt, Cairo, Egypt; 210 Zewail City of Science and Technology, Zewail, Egypt; 211 Purdue University, West Lafayette, USA; 212 Université de Haute Alsace, Mulhouse, France; 213 Tbilisi State University, Tbilisi, Georgia; 214 Erzincan Binali Yildirim University, Erzincan, Turkey; 215 CERN, European Organization for Nuclear Research, Geneva, Switzerland; 216 RWTH Aachen University, III. Physikalisches Institut A, Aachen, Germany; 217 University of Hamburg, Hamburg, Germany; 218 Department of Physics, Isfahan University of Technology, Isfahan, Iran; 219 Brandenburg University of Technology, Cottbus, Germany; 220 Skobeltsyn Institute of Nuclear Physics, Lomonosov Moscow State University, Moscow, Russia; 221 Institute of Physics, University of Debrecen, Debrecen, Hungary; 222 Physics Department, Faculty of Science, Assiut University, Assiut, Egypt; 223 MTA-ELTE Lendület CMS Particle and Nuclear Physics Group, Eötvös Loránd University, Budapest, Hungary; 224 Institute of Nuclear Research ATOMKI, Debrecen, Hungary; 225 IIT Bhubaneswar, Bhubaneswar, India; 226 Institute of Physics, Bhubaneswar, India; 227 G.H.G. Khalsa College, Punjab, India; 228 Shoolini University, Solan, India; 229 University of Hyderabad, Hyderabad, India; 230 University of Visva-Bharati, Santiniketan, India; 231 Indian Institute of Technology (IIT), Mumbai, India; 232 Deutsches Elektronen-Synchrotron, Hamburg, Germany; 233 Department of Physics, University of Science and Technology of Mazandaran, Behshahr, Iran; 234 INFN Sezione di Bari, Università di Bari, Politecnico di Bari, Bari, Italy; 235 Italian National Agency for New Technologies, Energy and Sustainable Economic Development, Bologna, Italy; 236 Centro Siciliano di Fisica Nucleare e di Struttura Della Materia, Catania, Italy; 237 Università di Napoli ’Federico II’, Naples, Italy; 238 Riga Technical University, Riga, Latvia; 239 Consejo Nacional de Ciencia y Tecnología, Mexico City, Mexico; 240 Warsaw University of Technology, Institute of Electronic Systems, Warsaw, Poland; 241 Institute for Nuclear Research, Moscow, Russia; 242 National Research Nuclear University ‘Moscow Engineering Physics Institute’ (MEPhI), Moscow, Russia; 243 St. Petersburg State Polytechnical University, St. Petersburg, Russia; 244 University of Florida, Gainesville, USA; 245 Imperial College, London, UK; 246 Moscow Institute of Physics and Technology, Moscow, Russia; 247 P.N. Lebedev Physical Institute, Moscow, Russia; 248 California Institute of Technology, Pasadena, USA; 249 Budker Institute of Nuclear Physics, Novosibirsk, Russia; 250 Faculty of Physics, University of Belgrade, Belgrade, Serbia; 251 Trincomalee Campus, Eastern University, Nilaveli, Sri Lanka; 252 INFN Sezione di Pavia, Università di Pavia, Pavia, Italy; 253 National and Kapodistrian University of Athens, Athens, Greece; 254 Universität Zürich, Zurich, Switzerland; 255 Stefan Meyer Institute for Subatomic Physics, Vienna, Austria; 256 Laboratoire d’Annecy-le-Vieux de Physique des Particules, IN2P3-CNRS, Annecy-le-Vieux, France; 257 Şırnak University, Sirnak, Turkey; 258 Department of Physics, Tsinghua University, Beijing, China; 259 Near East University, Research Center of Experimental Health Science, Nicosia, Turkey; 260 Beykent University, Istanbul, Turkey; 261 Istanbul Aydin University, Application and Research Center for Advanced Studies (App. & Res. Cent. for Advanced Studies), Istanbul, Turkey; 262 Mersin University, Mersin, Turkey; 263 Piri Reis University, Istanbul, Turkey; 264 Adiyaman University, Adiyaman, Turkey; 265 Ozyegin University, Istanbul, Turkey; 266 Izmir Institute of Technology, Izmir, Turkey; 267 Necmettin Erbakan University, Konya, Turkey; 268 Bozok Universitetesi Rektörlügü, Yozgat, Turkey; 269 Marmara University, Istanbul, Turkey; 270 Milli Savunma University, Istanbul, Turkey; 271 Kafkas University, Kars, Turkey; 272 Istanbul Bilgi University, Istanbul, Turkey; 273 Hacettepe University, Ankara, Turkey; 274 School of Physics and Astronomy, University of Southampton, Southampton, UK; 275 IPPP Durham University, Durham, UK; 276 Monash University, Faculty of Science, Clayton, Australia; 277 Bethel University, St. Paul, Minneapolis, USA; 278 Karamanoğlu Mehmetbey University, Karaman, Turkey; 279 Ain Shams University, Cairo, Egypt; 280 Bingol University, Bingol, Turkey; 281 Georgian Technical University, Tbilisi, Georgia; 282 Sinop University, Sinop, Turkey; 283 Mimar Sinan University, Istanbul, Turkey; 284 Nanjing Normal University Department of Physics, Nanjing, China; 285 Texas A&M University at Qatar, Doha, Qatar; 286 Kyungpook National University, Daegu, Korea; 287 CERN, 1211 Geneva 23, Switzerland

## Abstract

The production of Z boson pairs in proton–proton (${\mathrm{p}} {\mathrm{p}}$) collisions, ${{\mathrm{p}} {\mathrm{p}} \rightarrow ({\mathrm{Z}}/\gamma ^*)({\mathrm{Z}}/\gamma ^*) \rightarrow 2\ell 2\ell '}$, where ${\ell ,\ell ' = {\mathrm{e}}}$ or ${{\upmu }}$, is studied at a center-of-mass energy of 13$\,\text {TeV}$ with the CMS detector at the CERN LHC. The data sample corresponds to an integrated luminosity of 137$\,\text {fb}^{-1}$, collected during 2016–2018. The ${\mathrm{Z}} {\mathrm{Z}}$ production cross section, $\sigma _{\text {tot}} ({\mathrm{p}} {\mathrm{p}} \rightarrow {\mathrm{Z}} {\mathrm{Z}} ) = 17.4 \pm 0.3 \,\text {(stat)} \pm 0.5 \,\text {(syst)} \pm 0.4 \,\text {(theo)} \pm 0.3 \,\text {(lumi)} \text { pb}$, measured for events with two pairs of opposite-sign, same-flavor leptons produced in the mass region ${60< m_{\ell ^+\ell ^-} < 120\,\text {GeV}}$ is consistent with standard model predictions. Differential cross sections are also measured and agree with theoretical predictions. The invariant mass distribution of the four-lepton system is used to set limits on anomalous ${\mathrm{Z}} {\mathrm{Z}} {\mathrm{Z}}$ and ${{\mathrm{Z}} {\mathrm{Z}} \gamma }$ couplings.

## Introduction

Measurements of diboson production in proton–proton (${\mathrm{p}} {\mathrm{p}}$) collisions, such as Z boson pair (${\mathrm{Z}} {\mathrm{Z}}$) production, at the CERN LHC allow precision tests of the standard model (SM). In the SM, ${{\mathrm{Z}} {\mathrm{Z}} }$ production proceeds mainly through quark-antiquark *t*- and *u*-channel scattering diagrams. In calculations at higher orders in quantum chromodynamics (QCD), gluon-gluon fusion also contributes via box diagrams with quark loops. There are no tree-level contributions to ${{\mathrm{Z}} {\mathrm{Z}} }$ production from triple gauge boson vertices in the SM. Anomalous triple gauge couplings (aTGC) ${{\mathrm{Z}} {\mathrm{Z}} {\mathrm{Z}} }$ and ${{\mathrm{Z}} {\mathrm{Z}} \gamma }$ are introduced using an effective Lagrangian following Ref. [1]. In this parametrization, two ${{\mathrm{Z}} {\mathrm{Z}} {\mathrm{Z}} }$ and two ${{\mathrm{Z}} {\mathrm{Z}} \gamma }$ couplings are allowed by the electromagnetic gauge invariance and Lorentz invariance for on-shell ${{\mathrm{Z}}}$ bosons and are parametrized by two CP-violating (${f_4^{{\mathrm{V}}}}$) and two CP-conserving (${f_5^{{\mathrm{V}}}}$) parameters, where ${{{\mathrm{V}}} = ({\mathrm{Z}}, \gamma )}$. Nonzero aTGC values could be induced by new physics models such as supersymmetry [2]. The results can be also expressed in terms of parameters calculated within the effective field theory (EFT) framework, per convention used in Ref. [3] and references therein. In contrast to the anomalous couplings of electroweak (EW) vector bosons, the EFT framework allows an unambiguous calculation of loop effects and provides a simpler interpretation of the results than the aTGC framework.

Previous measurements of the production cross section for pairs of on-shell ${{\mathrm{Z}}}$ bosons at the LHC were performed by the CMS Collaboration with data sets corresponding to integrated luminosities of 5.1$\,\text {fb}^{-1}$ at $\sqrt{s} = 7\,\text {TeV}$ [4] and 19.6$\,\text {fb}^{-1}$ at $\sqrt{s} = 8\,\text {TeV}$ [5, 6] in the ${{\mathrm{Z}} {\mathrm{Z}} \rightarrow 2\ell 2\ell ^{\prime \prime }}$ and ${{\mathrm{Z}} {\mathrm{Z}} \rightarrow 2\ell 2\nu }$ decay channels, where ${\ell = {\mathrm{e}}}$ or ${{\upmu }}$ and ${\ell ^{\prime \prime } = {\mathrm{e}}, {\upmu }}$, or ${{\uptau }}$; and with an integrated luminosity of 2.6$\,\text {fb}^{-1}$  [7] and 35.9$\,\text {fb}^{-1}$  [8] at $\sqrt{s} = 13\,\text {TeV}$ in the ${{\mathrm{Z}} {\mathrm{Z}} \rightarrow 2\ell 2\ell ' }$ decay channel, where ${\ell ' = {\mathrm{e}}}$ or ${{\upmu }}$. All of the results agree with SM predictions. The ATLAS Collaboration reported similar results at $\sqrt{s} = 7$, 8, and 13$\,\text {TeV}$  [9–14], which also agree with SM predictions. These measurements are important to test predictions that were recently made available at next-to-next-to-leading order (NNLO) in QCD [15–17]. A comparison of these predictions with data for a range of center-of-mass energies provides an insight into the structure of the EW gauge sector of the SM.

This paper reports a study of the ${\mathrm{Z}} {\mathrm{Z}}$ production in the four-lepton decay channel (${{\mathrm{p}} {\mathrm{p}} \rightarrow 2\ell 2\ell ' }$, where ${2\ell }$ and ${2\ell '}$ indicate pairs of opposite-sign electrons or muons) at $\sqrt{s} = 13\,\text {TeV}$, with a data set corresponding to an integrated luminosity of $137{\,\text {fb}^{-1}}$ recorded in 2016–2018. Both ${{\mathrm{Z}}}$ bosons are selected to be on-shell, defined as the mass range 60–120$\,\text {GeV}$. Fiducial and total cross sections are measured, differential cross sections are presented as a function of different kinematic variables. The invariant mass distribution of the four-lepton system is used to search for anomalous ${{\mathrm{Z}} {\mathrm{Z}} {\mathrm{Z}} }$ and ${{\mathrm{Z}} {\mathrm{Z}} \gamma }$ couplings.

## The CMS detector

A detailed description of the CMS detector, together with a definition of the coordinate system used and the relevant kinematic variables, can be found in Ref. [18].

The central feature of the CMS apparatus is a superconducting solenoid of 6$\text { m}$ internal diameter, providing a magnetic field of 3.8$\text { T}$. Within the solenoid volume are a silicon pixel and strip tracker, a lead tungstate crystal electromagnetic calorimeter (ECAL), and a brass and scintillator hadron calorimeter, which provide coverage in pseudorapidity ${| \eta | < 1.479}$ in a cylindrical barrel and $1.479< | \eta | < 3.0$ in two endcap regions. Forward calorimeters extend the coverage provided by the barrel and endcap detectors to $|\eta | < 5.0$. Muons are detected in gas-ionization detectors embedded in the steel flux-return yoke outside the solenoid in the range $|\eta | < 2.4$, with detection planes made using three technologies: drift tubes, cathode strip chambers, and resistive-plate chambers.

Electron momenta are estimated by combining energy measurements in the ECAL with momentum measurements in the tracker. The momentum resolution for electrons with transverse momentum $p_{\mathrm {T}} \approx 45\,\text {GeV}$ from ${{\mathrm{Z}} \rightarrow {{\mathrm{e}}}{}^{+} {{\mathrm{e}}}{}^{-}}$ decays ranges from 1.7% for nonshowering electrons in the barrel region to 4.5% for showering electrons in the endcaps [19]. Matching muons to tracks identified in the silicon tracker results in a $p_{\mathrm {T}}$ resolution for muons with $20<p_{\mathrm {T}} < 100\,\text {GeV}$ of 1.3–2.0% in the barrel and better than 6% in the endcaps. The $p_{\mathrm {T}}$ resolution in the barrel is better than 10% for muons with $p_{\mathrm {T}}$ up to 1$\,\text {TeV}$  [20, 21].

Events of interest are selected using a two-tiered trigger system [22]. The first level, composed of custom hardware processors, uses information from the calorimeters and muon detectors to select events at a rate of around 100$\text { kHz}$ within a time interval of less than 4 $\upmu$s. The second level, known as the high-level trigger, consists of a farm of processors running a version of the full event reconstruction software optimized for fast processing, and reduces the event rate to around 1$\text { kHz}$ before data storage.

## Signal and background simulation

Several Monte Carlo (MC) samples are used in the analysis to optimize the selection, calculate the signal efficiency, and estimate background contamination. The pythia  8.226 and 8.230 [23, 24] packages are used for parton showering, hadronization, and the underlying event simulation with the CUETP8M1 tune [25] and the parton distribution function (PDF) NNPDF23_lo_as_0130 [26] for the 2016 data-taking period, and the CP5 tune [27] and the NNPDF 31_nnlo_as_0118 PDF for the 2017 and 2018 data-taking periods.

Signal events are generated with powheg 2.0 [24, 28–31] at next-to-leading order (NLO) in QCD for quark-antiquark (${{\mathrm{q}} {{\mathrm{q}}}}$) processes and leading order (LO) for quark-gluon processes. This includes ${{\mathrm{Z}} {\mathrm{Z}} }$, ${{\mathrm{Z}} \gamma ^*}$, ${{\mathrm{Z}}}$, $\gamma ^*\gamma ^*$ with a constraint of ${m_{\ell \ell '} > 4\,\text {GeV}}$ applied to all pairs of oppositely charged leptons at the generator level to avoid infrared divergences. The gluon-gluon loop-induced process, ${{\mathrm{g}} {\mathrm{g}} \rightarrow {\mathrm{Z}} {\mathrm{Z}} }$, is simulated at LO with mcfm  v7.0 [32]. It also includes interference with the SM Higgs off-shell production. The SM Higgs decay is modeled with jhugen 3.1.8 [33–35] at LO. The cross sections are scaled to correspond to cross section values calculated at NNLO in QCD for ${{\mathrm{q}} {{\mathrm{q}}} \rightarrow {\mathrm{Z}} {\mathrm{Z}} }$ [15] (with a *K* factor of 1.1) and at NLO in QCD for ${{\mathrm{g}} {\mathrm{g}} \rightarrow {\mathrm{Z}} {\mathrm{Z}} }$ [36] (*K* factor of 1.7). Electroweak ${\mathrm{Z}} {\mathrm{Z}}$ production in association with two jets is generated with MadGraph  [37] at LO. It amounts to approximately 1% of the total number of ${\mathrm{Z}} {\mathrm{Z}}$ events.

Simulated events for the irreducible background processes containing four prompt leptons in the final state, such as ${{{\mathrm{t}} {}{{\mathrm{t}}}} {\mathrm{Z}}}$, ${{\mathrm{W}} {\mathrm{W}} {\mathrm{Z}}}$, ${{\mathrm{W}} {\mathrm{Z}} {\mathrm{Z}}}$, and ${{\mathrm{Z}} {\mathrm{Z}} {\mathrm{Z}}}$, where the last three are combined and denoted as VVV, are generated with MadGraph 5_amc@nlo v2.4.2 [37] at NLO with zero or one outgoing partons in the matrix element calculation and merged with the parton shower using the FxFx scheme [38]. The same MC is used for ${{\mathrm{W}} {\mathrm{Z}}}$ simulation.

Event samples with aTGC contributions included are generated at LO with sherpa  v2.1.1 [39]. The distributions from the sherpa samples are normalized such that the total yield of the SM sample is the same as that of the powheg+mcfm sample. More details are discussed in Sect. 10.

The detector response is simulated using a detailed description of the CMS detector implemented with the Geant4 package [40]. The reconstruction in simulation and data uses the same algorithms. The simulated samples include additional interactions per bunch crossing, referred to as pileup. The simulated events are weighted so that the pileup distribution matches the data.

Results are also compared to fixed-order predictions produced via the Matrix framework [41], a parton-level MC generator that uses tree and one-loop amplitudes from OpenLoops 2 [42] and two-loop amplitudes from Ref. [43], capable of producing differential predictions at up to NNLO in QCD and NLO in EW, as implemented in Matrix  v2.0.0_beta1 [44]. The calculation is performed with the NNPDF31_nnlo_as_0118_luxqed [45] PDF set with dynamic renormalization ($\mu _{\mathrm {R}}$) and factorization scales ($\mu _{\mathrm {F}}$) set to the four lepton mass for the differential and fiducial predictions, and with fixed scale set to the nominal Z boson mass for the total cross section. The quark-induced processes are calculated at NNLO in QCD and NLO in EW. The gluon-induced contribution is calculated at NLO in QCD [46]. Photon-induced contributions are also included at up to NLO EW. The calculation uses massless leptons, which leads to a divergence at low dilepton mass. To avoid this divergence, we impose the requirement ${p_{\mathrm {T}} ^{\ell } > 5\,\text {GeV}}$ on the photon-induced component for total cross section predictions. With this condition, the photon-induced contribution is less than 1% of the total production rate. The quark-induced NNLO QCD and NLO EW contributions are combined multiplicatively, and the gluon- and photon-induced contributions are combined additively following the procedure described in Ref. [44]. The predictions reported here are consistent with those published in Refs. [15–17].

## Event reconstruction

Individual particles – electrons, muons, photons, charged and neutral hadrons – in each collision event are identified and reconstructed with the CMS particle-flow (PF) algorithm [47] from a combination of signals from all subdetectors. Reconstructed electrons [19] and muons [20] are considered as lepton candidates if they have ${p_{\mathrm {T}} ^{{\mathrm{e}}} > 7\,\text {GeV}}$ and ${|\eta ^{{\mathrm{e}}} | < 2.5}$ or ${p_{\mathrm {T}} ^{{\upmu }} > 5\,\text {GeV}}$ and ${|\eta ^{{\upmu }} | < 2.4}$.

Lepton candidates are also required to originate from the primary vertex, defined as the reconstructed ${\mathrm{p}} {\mathrm{p}}$ interaction vertex with the largest value of summed physics object $p_{\mathrm {T}} ^2$. The physics objects used in the primary vertex definition are the objects returned by a jet-finding algorithm [48, 49] applied to all charged tracks associated with the vertex. The distance of closest approach between each lepton track and the primary vertex is required to be less than 0.5$\text { cm}$ in the plane transverse to the beam axis, and less than 1$\text { cm}$ in the direction along the beam axis. Furthermore, the significance of the three-dimensional impact parameter relative to the primary vertex, $\mathrm {SIP_{3D}}$, is required to satisfy $\mathrm {SIP_{3D}} \equiv | \mathrm {IP} / \sigma _\mathrm {IP} | < 4$ for each lepton, where $\mathrm {IP}$ is the distance of closest approach of each lepton track to the primary vertex and $\sigma _\mathrm {IP}$ is its associated uncertainty.

Lepton candidates are required to be isolated from other particles in the event. The relative isolation is defined as

1 $$\begin{aligned} R_\text {iso}= & {} \left[ \sum _{\begin{array}{c} \text {charged} \\ \text {hadrons} \end{array}} p_{\mathrm {T}} \, + \, \max \left( 0, \sum _{\begin{array}{c} \text {neutral} \nonumber \\ \text {hadrons} \end{array}} \right. \right. p_{\mathrm {T}} \\&\left. \left. + \, \sum _{\text {photons}} \!\! p_{\mathrm {T}} \, - \, p_{\mathrm {T}} ^{\mathrm {PU}} \right) \right] \bigg / p_{\mathrm {T}} ^{\ell }, \end{aligned}$$

where the sums run over the charged and neutral hadrons, and photons identified by the PF algorithm, in a cone defined by $\varDelta R \equiv \sqrt{\smash [b]{\left( \varDelta \eta \right) ^2 + \left( \varDelta \varphi \right) ^2}} < 0.3$ around the lepton momentum direction, where $\varphi$ is the azimuthal angle in radians. To minimize the contribution of charged particles from pileup to the isolation calculation, charged hadrons are included only if they originate from the primary vertex. The contribution of neutral particles from pileup is $p_{\mathrm {T}} ^{\mathrm {PU}}$. For electrons, $p_{\mathrm {T}} ^{\mathrm {PU}}$ is evaluated with the “jet area” method described in Ref. [50]; for muons, it is half of the summed $p_{\mathrm {T}}$ of all charged particles in the cone originating from pileup vertices. The average factor of one half accounts for the expected ratio of neutral to charged particle production in hadronic interactions. A lepton is considered isolated if $R_\text {iso} < 0.35$.

The lepton reconstruction, identification, and isolation efficiencies are measured with a “tag-and-probe” technique [51] applied to a sample of ${{\mathrm{Z}} \rightarrow \ell ^+\ell ^-}$ data events. The measurements are performed in several bins of ${p_{\mathrm {T}} ^{\ell }}$ and ${|\eta ^\ell |}$. The electron reconstruction and selection efficiency in the ECAL barrel (endcaps) varies from about 85 (77)% at ${p_{\mathrm {T}} ^{{\mathrm{e}}} \approx 10\,\text {GeV}}$ to about 95 (89)% for ${p_{\mathrm {T}} ^{{\mathrm{e}}} \ge 20\,\text {GeV}}$, whereas in the barrel-endcap transition region this efficiency is about 85% averaged over electrons with ${p_{\mathrm {T}} ^{{\mathrm{e}}} > 7\,\text {GeV}}$. The muons are reconstructed and identified with efficiencies above ${\sim }98\%$ within ${|\eta ^{{\upmu }} | < 2.4}$.

## Event selection

The primary triggers for this analysis require the presence of a pair of loosely isolated leptons of the same or different flavors [22]. The highest $p_{\mathrm {T}}$ lepton must have ${p_{\mathrm {T}} ^{\ell } > 17\,\text {GeV}}$, and the subleading lepton must have ${p_{\mathrm {T}} ^{{\mathrm{e}}} > 12\,\text {GeV}}$ if it is an electron or ${p_{\mathrm {T}} ^{{\upmu }} > 8\,\text {GeV}}$ if it is a muon. The tracks of the triggering leptons are required to originate within 2 mm of each other in the plane transverse to the beam axis. Triggers requiring a triplet of lower-$p_{\mathrm {T}}$ leptons with no isolation criteria, or a single high-$p_{\mathrm {T}}$ electron or muon, are also used. An event is accepted if it passes any trigger regardless of the decay channel. The total trigger efficiency for events within the acceptance of this analysis is greater than 98%.

The four-lepton candidate selection is based on the one used in the recent CMS Higgs boson measurement  [52]. A signal event must contain at least two ${{\mathrm{Z}}/\gamma ^{*}}$ candidates, each formed from an oppositely charged pair of isolated electron or muon candidates. Among the four leptons, the highest $p_{\mathrm {T}}$ lepton must have $p_{\mathrm {T}} > 20\,\text {GeV}$, and the second-highest $p_{\mathrm {T}}$ lepton must have ${p_{\mathrm {T}} ^{{\mathrm{e}}} > 12\,\text {GeV}}$ if it is an electron or ${p_{\mathrm {T}} ^{{\upmu }} > 10\,\text {GeV}}$ if it is a muon. All leptons are required to be separated from each other by ${\varDelta R \left( \ell _1, \ell _2 \right) > 0.02}$, and electrons are required to be separated from muons by ${\varDelta R \left( {\mathrm{e}}, \mu \right) > 0.05}$.

Within each event, all permutations of leptons giving a valid pair of ${{\mathrm{Z}}/\gamma ^{*}}$ candidates are considered separately. Within each four-lepton candidate, the dilepton candidate with an invariant mass closest to 91.2$\,\text {GeV}$, taken as the nominal ${{\mathrm{Z}}}$ boson mass [53], is denoted ${{\mathrm{Z}} _1}$ and is required to have a mass greater than 40$\,\text {GeV}$. The other dilepton candidate is denoted ${{\mathrm{Z}} _2}$ and is required to have a mass greater than 4$\,\text {GeV}$. Both $m_{Z_1}$ and $m_{Z_2}$ are required to be less than 120$\,\text {GeV}$. All pairs of oppositely charged leptons in the four-lepton candidate are required to have ${m_{\ell \ell '} > 4\,\text {GeV}}$ regardless of their flavor. In the rare case of further ambiguity, which occurs in less than 0.5% of events when five or more passing lepton candidates are found, the ${{\mathrm{Z}} _2}$ candidate that maximizes the scalar $p_{\mathrm {T}}$ sum of the four leptons is chosen.

The ${{\mathrm{p}} {\mathrm{p}} \rightarrow {\mathrm{Z}} {\mathrm{Z}} }$ cross section is measured using events where both ${m_{{\mathrm{Z}} _1}}$ and ${m_{{\mathrm{Z}} _2}}$ are greater than 60$\,\text {GeV}$. Decays of the ${{\mathrm{Z}}}$ bosons to ${{\uptau }}$ leptons with subsequent decays to electrons and muons are heavily suppressed by the requirements on lepton $p_{\mathrm {T}}$, and the contribution of such events is less than 0.5% of the total ${{\mathrm{Z}} {\mathrm{Z}} }$ yield. If these events pass the selection requirements of the analysis, they are considered signal, although they are not considered at generator level in the cross section measurement procedure. Thus, the correction for possible $\tau$ decays is included in the efficiency calculation.

## Background estimation

The requirement of four well-reconstructed and isolated lepton candidates strongly suppresses any background; therefore this analysis has very low background contributions, dominated by ${{\mathrm{Z}}}$ boson and ${{\mathrm{W}} {\mathrm{Z}}}$ diboson production in association with jets, and by t t production. In a small fraction of cases, particles from jet fragmentation satisfy both lepton identification and isolation criteria, and thus are misidentified as signal leptons. This background is estimated using control data samples, as decribed below.

The probability for such objects to be selected is measured from a sample of ${{\mathrm{Z}} +\ell _\text {candidate}}$ events, where ${{\mathrm{Z}}}$ denotes a pair of oppositely charged, same-flavor leptons that pass all analysis requirements and satisfy ${| m_{\ell ^+\ell ^-} - m_{{\mathrm{Z}}} | < 10\,\text {GeV}}$, where ${m_{{\mathrm{Z}}}}$ is the nominal ${{\mathrm{Z}}}$ boson mass. Each event in this sample must have exactly one additional object ${\ell _\text {candidate}}$ that passes relaxed identification requirements with no isolation requirements applied. The misidentification probability for each lepton flavor, measured in bins of lepton candidate $p_{\mathrm {T}}$ and $\eta$, is defined as the ratio between the number of candidates that pass the final isolation and identification requirements and the total number of candidates in the sample. The number of ${{\mathrm{Z}} +\ell _\text {candidate}}$ events is corrected for the contamination from ${{\mathrm{W}} {\mathrm{Z}}}$ production and for ${{\mathrm{Z}} {\mathrm{Z}} }$ events in which one lepton is not reconstructed. These events have a third genuine, isolated lepton that must be excluded from the misidentification probability calculation. The ${{\mathrm{W}} {\mathrm{Z}}}$ contamination is suppressed by requiring the missing transverse momentum $p_{\mathrm {T}} ^\text {miss}$ to be below 25$\,\text {GeV}$. The $p_{\mathrm {T}} ^\text {miss}$ is defined as the magnitude of the missing transverse momentum vector ${\mathbf {p}}_{\mathrm {T}}^{\text {miss}}$, the projection onto the plane transverse to the beams of the negative vector sum of the momenta of all reconstructed PF candidates in the event, corrected for the jet energy scale. The transverse mass, calculated as ${m_{\mathrm {T}} \equiv \sqrt{\smash [b]{(p_{\mathrm {T}} ^\ell + p_{\mathrm {T}} ^\text {miss})^2 - ({\mathbf {p}}_{\mathrm {T}} ^{\ell } + {\mathbf {p}}_{\mathrm {T}}^{\text {miss}})^2}}}$, is required to be less than 30$\,\text {GeV}$. The residual contribution of ${{\mathrm{W}} {\mathrm{Z}}}$ and ${{\mathrm{Z}} {\mathrm{Z}} }$ events, which can be up to a few percent of the events with ${\ell _\text {candidate}}$ passing all selection criteria, is estimated from simulation and subtracted.

To account for all sources of background events, two control samples are used to estimate the number of background events in the signal regions. Both are defined as samples that contain events with a dilepton candidate satisfying all requirements (${{\mathrm{Z}} _1}$) and two additional lepton candidates ${\ell ^{+}\ell ^{-}}$. In one control sample, enriched in ${{\mathrm{W}} {\mathrm{Z}}}$ events, one ${\ell }$ candidate is required to satisfy the full identification and isolation criteria and the other must fail the full criteria and instead satisfy only the relaxed ones; in the other, enriched in ${{\mathrm{Z}}}$+jets events, both ${\ell }$ candidates must satisfy the relaxed criteria, but fail the full criteria. The additional leptons must have opposite charges and the same flavor (${{\mathrm{e}} ^{\pm }{\mathrm{e}} ^{\mp }}$ and ${{\upmu } ^{\pm }{\upmu } ^{\mp }}$). From this set of events, the expected number of background events in the signal region, denoted “${{\mathrm{Z}}}$+X” in the figures, is obtained by scaling the number of observed ${{\mathrm{Z}} _1+\ell ^{+}\ell ^{-}}$ events by the misidentification probability for each lepton failing the selection. The procedure is described in more detail in Ref. [54].

In addition to this reducible background, which contributes to approximately 1–2% of the ${\mathrm{Z}} {\mathrm{Z}}$ events, the ${{{\mathrm{t}} {}{{\mathrm{t}}}} {\mathrm{Z}}}$ and VVV processes with four prompt leptons are estimated from simulated samples to be around 1–1.5% of the expected ${{\mathrm{Z}} {\mathrm{Z}} \rightarrow 2\ell 2\ell '}$ yield. The total background contributions to the ${{\mathrm{Z}} {\mathrm{Z}} \rightarrow 2\ell 2\ell '}$ signal regions are summarized in Sect. 8.

## Systematic uncertainties

The major sources of systematic uncertainty and their effect on the measured cross sections are summarized in Table 1. The lepton identification, isolation, and track reconstruction efficiencies in simulation are corrected with scaling factors derived with a tag-and-probe method and applied as a function of lepton $p_{\mathrm {T}}$ and $\eta$. To estimate the uncertainties associated with the tag-and-probe technique, the total yield is recomputed with the scaling factors varied up and down by the tag-and-probe fit uncertainties. The uncertainties associated with the lepton efficiency in the ${{\mathrm{Z}} {\mathrm{Z}} \rightarrow 2\ell 2\ell '}$ signal regions are 5% in the ${4{\mathrm{e}}}$, 3% in the ${2{\mathrm{e}} 2\mu }$, and 2% in the $4\mu$ final states.

In both data and simulated event samples, trigger efficiencies are evaluated with a tag-and-probe technique. The ratio of the trigger efficiency estimated using data to the one estimated with simulation is applied to simulated events, and the size of the resulting change in the expected yield is taken as the uncertainty in the determination of the trigger efficiency. This uncertainty is around 1–2% of the final estimated yield.

**Table 1:** The contributions of each source of systematic uncertainty in the cross section measurements. The integrated luminosity uncertainty, and the PDF and scale uncertainties, are considered separately. All other uncertainties are added in quadrature into a single systematic uncertainty. Uncertainties that vary by decay channel are listed as ranges

Uncertainty	Range of values
Lepton efficiency	2–5%
Trigger efficiency	1–2%
Background	0.6–1.3%
Pileup	1%
$\mu _\mathrm {R}$, $\mu _\mathrm {F}$	1%
PDF	1%
NNLO/NLO corrections	1%
Integrated luminosity	2.5% (2016), 2.3% (2017),
	2.5% (2018)

**Fig. 1:**
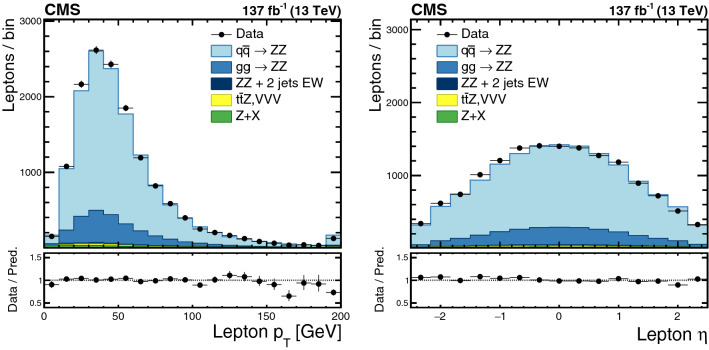
Distributions of (left) transverse momentum and (right) pseudorapidity for individual leptons. Points represent the data with error bars showing the statistical uncertainties, histograms the expected SM predictions and reducible background estimated from data. The $p_{\mathrm {T}}$ distributions includes overflow in the last bin

The largest uncertainty in the estimated background yield arises from differences in sample composition between the ${{\mathrm{Z}} +\ell _\text {candidate}}$ control sample used to calculate the lepton misidentification probability and the ${{\mathrm{Z}} +\ell ^+\ell ^-}$ control sample. An additional uncertainty arises from the limited number of events in the ${{\mathrm{Z}} +\ell _\text {candidate}}$ sample. A systematic uncertainty of 40% is applied to the lepton misidentification probability to cover both effects. Its impact varies by channel, but is of the order of 1% of the total expected yield.

The modeling of pileup relies on the total inelastic ${\mathrm{p}} {\mathrm{p}}$ cross Sect. [55]. The pileup uncertainty is evaluated by varying this cross section up and down by 5%.

**Fig. 2:**
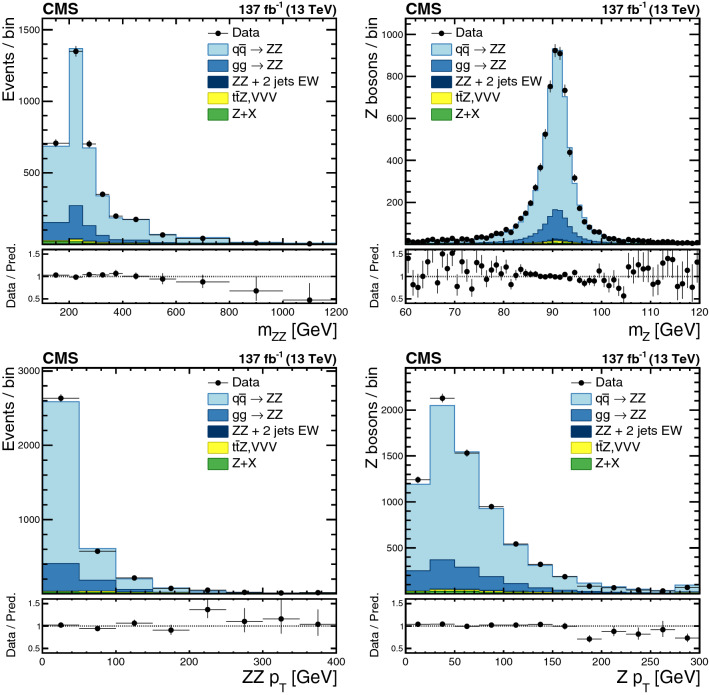
Distributions of (upper left) ${m_{{\mathrm{Z}} {\mathrm{Z}} }}$ for ${\mathrm{Z}} {\mathrm{Z}}$ events with ${60< m_{{\mathrm{Z}} _1, {\mathrm{Z}} _2} < 120 \,\text {GeV}}$; (upper right) mass of selected Z boson candidates; (lower left) transverse momentum of the ${{\mathrm{Z}} {\mathrm{Z}} }$ system; (lower right) transverse momentum of individual Z boson candidates. Points represent the data with error bars showing the statistical uncertainties, histograms the expected SM predictions and reducible background estimated from data. All $p_{\mathrm {T}}$ and ${m_{{\mathrm{Z}} {\mathrm{Z}} }}$ distributions include overflow in the last bin

Uncertainties because of factorization ($\mu _\mathrm {F}$) and renormalization ($\mu _\mathrm {R}$) scale choices on the ${{\mathrm{Z}} {\mathrm{Z}} \rightarrow 2\ell 2\ell '}$ acceptance are evaluated with powheg+mcfm by varying $\mu _\mathrm {F}$ and $\mu _\mathrm {R}$ up and down by a factor of two with respect to the default values ${\mu _\mathrm {F} = \mu _\mathrm {R} = m_{{\mathrm{Z}} {\mathrm{Z}} }}$, where ${m_{{\mathrm{Z}} {\mathrm{Z}} }}$ is the invariant mass of the ${\mathrm{Z}} {\mathrm{Z}}$ system. All combinations are considered except those in which $\mu _\mathrm {F}$ and $\mu _\mathrm {R}$ differ by a factor of four. Parametric uncertainties (PDF+$\alpha _\mathrm {S}$) are evaluated according to the PDF4LHC prescription [56] in the acceptance calculation, and with NNPDF3.0 [57] in the cross section calculations. An additional theoretical uncertainty arises from scaling the powheg ${{\mathrm{q}} {{\mathrm{q}}} \rightarrow {\mathrm{Z}} {\mathrm{Z}} }$ simulated sample from its NLO cross section to the NNLO prediction, and the mcfm ${{\mathrm{g}} {\mathrm{g}} \rightarrow {\mathrm{Z}} {\mathrm{Z}} }$ samples from their LO cross sections to the NLO predictions. The change in the acceptance corresponding to this scaling procedure is about 1%.

The uncertainty in the integrated luminosity of the data samples is 2.5% (2016) [58], 2.3% (2017) [59], and 2.5% (2018) [60]. Since the luminosity uncertainty contains a significant uncorrelated portion, the relative luminosity uncertainty of the whole sample is smaller than for each individual year.

The same uncertainties are valid for both total and differential cross section measurements, but for the differential one there is also an additional uncertainty related to the unfolding procedure described in Sect. 9. It is estimated using MadGraph 5_amc@nlo instead of powheg+mcfm in unfolding. The unfolding uncertainty is included in the results and plots together with other uncertainites, but its effect is small compared to the statistical uncertainties of the measurement.

**Table 2:** Observed and expected prefit yields of ${{\mathrm{Z}} {\mathrm{Z}} }$ events, and estimated yields of background events, shown for each final state and combined. The statistical (first) and systematic (second) uncertainties are presented

Process	${{\mathrm{e}} {\mathrm{e}} {\mathrm{e}} {\mathrm{e}}}$	${{\mathrm{e}} {\mathrm{e}} {\upmu } {\upmu }}$	${{\upmu } {\upmu } {\upmu } {\upmu }}$	${2\ell 2\ell '}$
	2016			
Background	6.7 ± 0.6 ± 1.8	11.4 ± 0.8 ± 1.9	5.5 ± 0.5 ± 0.9	23.6 ± 1.1 ± 4.1
Signal	167.7 ± 1.0 ± 10.0	434.2 ± 1.6 ± 17.3	273.3 ± 1.3 ± 8.2	875.2 ± 2.3 ± 31.1
Total expected	174.4 ± 1.2 ± 10.4	445.6 ± 1.8 ± 17.7	278.8 ± 1.4 ± 8.4	898.8 ± 2.6 ± 32.0
Data	176	478	296	950
	2017			
Background	6.3 ± 0.4 ± 1.5	12.1 ± 0.8 ± 1.8	7.9 ± 0.7 ± 1.6	26.3 ± 1.2 ± 4.5
Signal	200.8 ± 0.3 ± 12.0	511.7 ± 0.6 ± 20.4	322.5 ± 0.5 ± 9.6	1035.0 ± 0.8 ± 36.9
Total expected	207.1 ± 0.6 ± 12.4	523.8 ± 1.0 ± 20.9	330.4 ± 0.8 ± 9.9	1061.3 ± 1.4 ± 38.0
Data	193	540	328	1061
	2018			
Background	9.9 ± 0.6 ± 2.5	23.2 ± 1.1 ± 4.2	15.6 ± 1.1 ± 4.0	48.7 ± 1.7 ± 9.7
Signal	305.2 ± 0.4 ± 18.2	758.5 ± 0.8 ± 30.1	467.3 ± 0.6 ± 13.9	1531.0 ± 1.0 ± 54.7
Total expected	315.1 ± 0.8 ± 18.7	781.7 ± 1.4 ± 31.1	482.9 ± 1.3 ± 14.8	1579.7 ± 2.0 ± 56.6
Data	309	797	480	1586

## Cross section measurement

The $p_{\mathrm {T}}$ and $\eta$ distributions for individual leptons are shown in Fig. 1. Both distributions contain four leptons per event. The invariant mass of the ${\mathrm{Z}} {\mathrm{Z}}$ system, the individual mass of reconstructed Z boson candidates in the ${\mathrm{Z}} {\mathrm{Z}}$ events, and their corresponding $p_{\mathrm {T}}$ distributions are shown in Fig. 2. The last bins in ${m_{{\mathrm{Z}} {\mathrm{Z}} }}$ and all $p_{\mathrm {T}}$ distributions contain events from the overflow. The ${m_{\mathrm{Z}}}$ and Z $p_{\mathrm {T}}$ distributions contain two Z candidates for each event. These distributions are shown for data and simulated events to demonstrate comparisons with SM expectations. The signal expectations include contributions from ${{\mathrm{Z}} {\mathrm{Z}} }$ production shown separately for ${{\mathrm{q}} {{\mathrm{q}}} \rightarrow {\mathrm{Z}} {\mathrm{Z}} }$, ${{\mathrm{g}} {\mathrm{g}} \rightarrow {\mathrm{Z}} {\mathrm{Z}} }$, and EW ${\mathrm{Z}} {\mathrm{Z}}$ processes in all figures and combined as “Signal” in Table 2. The EW ${\mathrm{Z}} {\mathrm{Z}}$ production contributes to approximately 1% of the total number of ${\mathrm{Z}} {\mathrm{Z}}$ events.

**Table 3:** Measured fiducial cross section for each data sample and combined. The first uncertainty is statistical, the second is experimental systematic, and the third is associated with the integrated luminosity

Year	Fiducial cross section, fb
2016	$42.0 \pm 1.4 \,\text {(stat)} \pm 1.3 \,\text {(syst)} _{-1.0}^{+1.1} \,\text {(lumi)}$
2017	$39.6 \pm 1.2 \,\text {(stat)} _{-1.2}^{+1.3} \,\text {(syst)} _{-0.9}^{+1.0} \,\text {(lumi)}$
2018	$39.7 \pm 1.0 \,\text {(stat)} _{-1.1}^{+1.3} \,\text {(syst)} \pm 1.0 \,\text {(lumi)}$
Combined	$40.5 \pm 0.7 \,\text {(stat)} \pm 1.1 \,\text {(syst)} \pm 0.7 \,\text {(lumi)}$

**Table 4:** Measured total ${\sigma ({\mathrm{p}} {\mathrm{p}} \rightarrow {\mathrm{Z}} {\mathrm{Z}} )}$ cross section for each data sample and combined. The first uncertainty is statistical, the second is experimental systematic, the third is theoretical systematic. The fourth uncertainty is associated with the integrated luminosity

Year	Total cross section, pb
2016	$18.1 \pm 0.6 \,\text {(stat)} _{-0.5}^{+0.6} \,\text {(syst)} \pm 0.4 \,\text {(theo)} _{-0.4}^{+0.5} \,\text {(lumi)}$
2017	$17.0 \pm 0.5 \,\text {(stat)} _{-0.5}^{+0.6} \,\text {(syst)} \pm 0.4 \,\text {(theo)} \pm 0.4 \,\text {(lumi)}$
2018	$17.1 \pm 0.4 \,\text {(stat)} \pm 0.5 \,\text {(syst)} \pm 0.4 \,\text {(theo)} \pm 0.4 \,\text {(lumi)}$
Combined	$17.4 \pm 0.3 \,\text {(stat)} \pm 0.5 \,\text {(syst)} \pm 0.4 \,\text {(theo)} \pm 0.3 \,\text {(lumi)}$

The irreducible background, which amounts to 1–1.5% of the total ${\mathrm{Z}} {\mathrm{Z}}$ yield, and reducible background are combined as “Background” in Table 2. The total background in this analysis is $\approx 3\%$. The estimated yields agree well with the measured ones. The individual distributions are well described, except the ${m_{{\mathrm{Z}} {\mathrm{Z}} }}$ distribution at high values of invariant masses and the ${p_{\mathrm {T}} ^{\mathrm{Z}}}$ distribution at high values of $p_{\mathrm {T}}$. These are regions where the EW corrections may become important and will be discussed later in Sect. 10.

The measured yields are used to evaluate the ${{\mathrm{Z}} {\mathrm{Z}} }$ production cross section in the fiducial phase space. The signal acceptance is evaluated from simulation and corrected for each individual lepton flavor in bins of $p_{\mathrm {T}}$ and $\eta$ using factors obtained with the tag-and-probe technique. To include all final states in the cross section calculation, a simultaneous fit to the number of observed events in all decay channels is performed. The likelihood is composed as a combination of individual channel likelihoods for the signal and background hypotheses with the statistical and systematic uncertainties treated as scaling nuisance parameters. The combination of various data-taking periods is performed treating the theoretical uncertainties as fully correlated among various periods, whereas the experimental uncertainties are either correlated or uncorrelated, depending on their origin.

**Fig. 3:**
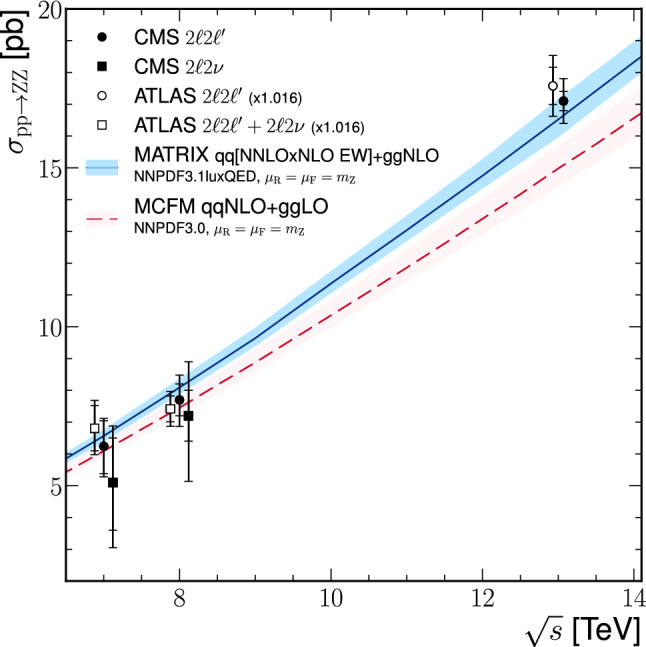
The total ${\mathrm{Z}} {\mathrm{Z}}$ cross section as a function of the proton–proton center-of-mass energy. Results from the CMS [4, 5] and ATLAS [9, 10, 14] experiments are compared to predictions from Matrix at NNLO in QCD and NLO in EW, and mcfm at NLO in QCD. The mcfm prediction also includes gluon-gluon initiated production at LO in QCD. The predictions use NNPDF31_nnlo_as_0118_luxqed and NNPDF3.0 PDF sets, respectively, and fixed factorization and renormalization scales ${\mu _\mathrm {F} = \mu _\mathrm {R} = m_{{\mathrm{Z}}}}$. Details of the calculations and uncertainties are given in the text. The ATLAS measurements were performed with a ${{\mathrm{Z}}}$ boson mass window of 66–116$\,\text {GeV}$, instead of 60–120$\,\text {GeV}$ used by CMS, and are corrected for the resulting 1.6% difference in acceptance. Measurements at the same center-of-mass energy are shifted slightly along the horizontal axis for clarity

The fiducial phase space for the ${{\mathrm{Z}} {\mathrm{Z}} \rightarrow 2\ell 2\ell '}$ cross section measurement is defined as: ${p_{\mathrm {T}} ^{\ell _1} > 20\,\text {GeV}}$, ${p_{\mathrm {T}} ^{\ell _2} > 10\,\text {GeV}}$, ${p_{\mathrm {T}} ^{\ell _{3,4}} > 5\,\text {GeV}}$, ${|\eta ^{\ell } | < 2.5}$, ${m_{2\ell } > 4\,\text {GeV}}$ (any opposite-sign same-flavor pair), ${60< m_{{\mathrm{Z}} _1}, m_{{\mathrm{Z}} _2} < 120\,\text {GeV}}$. The generator-level leptons used for the fiducial cross section calculation are “dressed” by adding the momenta of generator-level photons within ${\varDelta R\left( \ell ,\gamma \right) < 0.1}$ from the lepton momenta directions.

The measured ${{\mathrm{Z}} {\mathrm{Z}} }$ fiducial cross section presented in Table 3 can be compared to $39.3^{+0.8}_{-0.7} \pm 0.6\text { fb}$ calculated with powheg+mcfm using the same settings as the simulated samples with *K* factors applied. The first uncertainty corresponds to the factorization and renormalization scales and the second to PDF, as described above. The powheg calculations used dynamic factorization and renormalization scales ${\mu _\mathrm {F} = \mu _\mathrm {R} = m_{2\ell 2\ell '}}$, whereas the contribution from mcfm is computed with dynamic scales ${\mu _\mathrm {F} = \mu _\mathrm {R} = 0.5 m_{2\ell 2\ell '}}$. It can also be compared to the prediction from Matrix  v2.0.0_beta1 of $38.0^{+1.1}_{-1.0}$. The uncertainty in the Matrix prediction includes only the uncertainty due to the variation of $\mu _\mathrm {F}$ and $\mu _\mathrm {R}$.

The total ${{\mathrm{Z}} {\mathrm{Z}} }$ production cross section for both dileptons produced in the mass range 60–120$\,\text {GeV}$ and ${m_{\ell ^+\ell ^{\prime -}} > 4\,\text {GeV}}$ is presented in Table 4. The nominal branching fraction ${\mathcal {B}({\mathrm{Z}} \rightarrow \ell ^+\ell ^-) = 0.03366}$ is used [53]. The measured total cross section can be compared to the theoretical value of $16.9^{+0.6}_{-0.5} \pm 0.2\text { pb}$, calculated from powheg+mcfm with the same settings that is used for ${\sigma _{\mathrm {fid}} ({\mathrm{p}} {\mathrm{p}} \rightarrow {\mathrm{Z}} {\mathrm{Z}} \rightarrow 2\ell 2\ell ')}$. It can also be compared to $16.5^{+0.6}_{-0.5}$ $\text { pb}$, calculated with Matrix  v2.0.0_beta1, or $15.0^{+0.7}_{-0.6} \pm 0.2$ $\text { pb}$, calculated with mcfm at NLO in QCD with additional contributions from LO ${{\mathrm{g}} {\mathrm{g}} \rightarrow {\mathrm{Z}} {\mathrm{Z}} }$ diagrams and with the NLO NNPDF3.0 PDF set and fixed factorization and renormalization scales set to ${\mu _\mathrm {F} = \mu _\mathrm {R} = m_{{\mathrm{Z}}}}$.

**Fig. 4:**
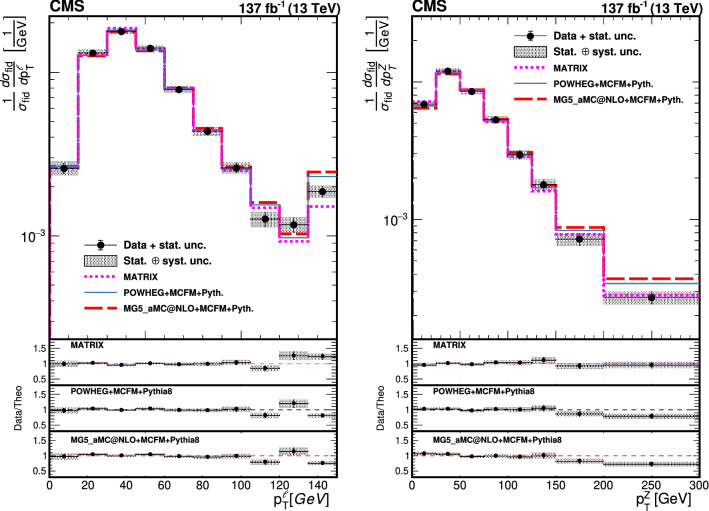
Differential cross sections normalized to the fiducial cross section for the combined ${4{\mathrm{e}}}$, ${2{\mathrm{e}} 2{\upmu }}$, and ${4{\upmu }}$ decay channels as a function of $p_{\mathrm {T}}$ for (left) all leptons, (right) all ${{\mathrm{Z}}}$ bosons in the event. The points represent the unfolded data with error bars showing the statistical uncertainties, the shaded histogram the powheg+mcfm ${\mathrm{Z}} {\mathrm{Z}}$ predictions, and the dashed curves correspond to the results of the Matrix and MadGraph 5_amc@nlo+mcfm calculations. The three lower panels represent the ratio of the measured cross section to the expected distributions from Matrix, powheg+mcfm and MadGraph 5_amc@nlo+mcfm. The shaded areas in all the panels represent the full uncertainties calculated as the quadratic sum of the statistical and systematic uncertainties, whereas the crosses represent only the statistical uncertainties

**Fig. 5:**
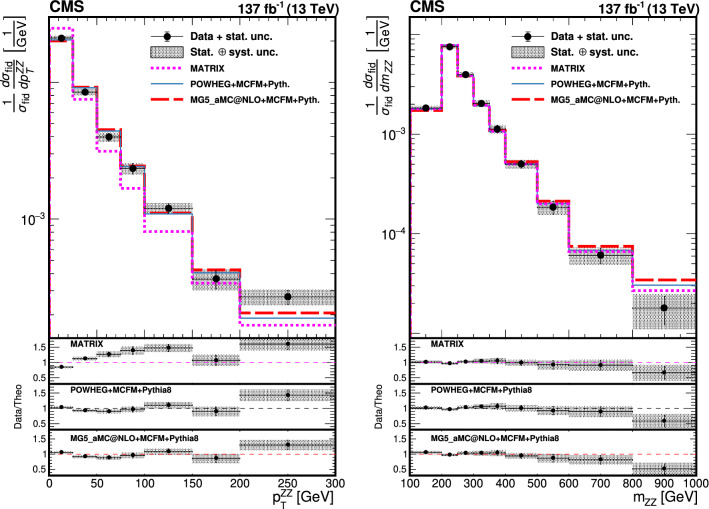
Differential cross sections normalized to the fiducial cross section for the combined ${4{\mathrm{e}}}$, ${2{\mathrm{e}} 2{\upmu }}$, and ${4{\upmu }}$ decay channels as a function of (left) $p_{\mathrm {T}}$ of the ${{\mathrm{Z}} {\mathrm{Z}} }$ system, (right) the invariant mass of the ${\mathrm{Z}} {\mathrm{Z}}$ system. The points represent the unfolded data with error bars showing the statistical uncertainties, shaded histogram the powheg+mcfm ${\mathrm{Z}} {\mathrm{Z}}$ predictions, and the dashed curves correspond to the results of the Matrix and MadGraph 5_amc@nlo+mcfm calculations. The three lower panels represent the ratio of the measured cross section to the expected distributions from Matrix, powheg+mcfm and MadGraph 5_amc@nlo+mcfm. The shaded areas in all the panels represent the full uncertainties calculated as the quadratic sum of the statistical and systematic uncertainties, whereas the crosses represent only the statistical uncertainties

**Fig. 6:**
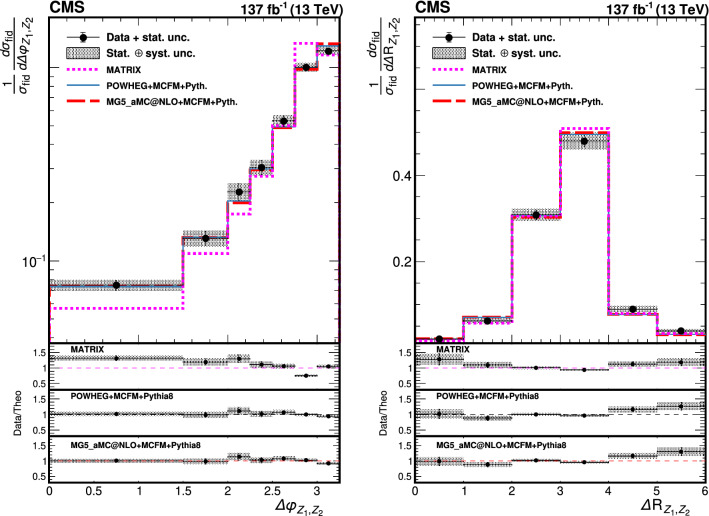
Differential cross sections normalized to the fiducial cross section for the combined ${4{\mathrm{e}}}$, ${2{\mathrm{e}} 2{\upmu }}$, and ${4{\upmu }}$ decay channels as a function of the azimuthal (left) and $\varDelta$*R* (right) separation of the two ${{\mathrm{Z}}}$ bosons. The points represent the unfolded data with error bars showing the statistical uncertainties, the shaded histogram the powheg+mcfm ${\mathrm{Z}} {\mathrm{Z}}$ predictions, and the dashed curves correspond to the results of the Matrix and MadGraph 5_amc@nlo+mcfm calculations. The three lower panels represent the ratio of the measured cross section to the expected distributions from Matrix, powheg+mcfm and MadGraph 5_amc@nlo+mcfm. The shaded areas in all the panels represent the full uncertainties calculated as the quadratic sum of the statistical and systematic uncertainties, whereas the crosses represent only the statistical uncertainties

**Fig. 7:**
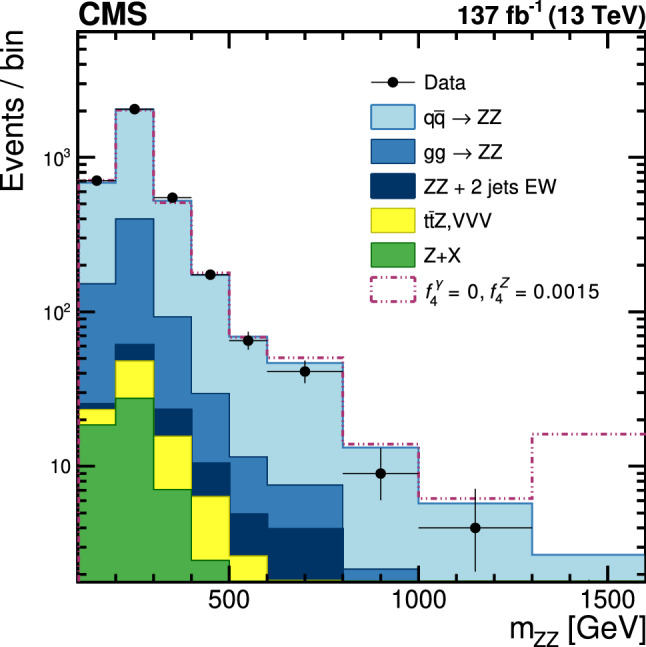
Distribution of the reconstructed ${\mathrm{Z}} {\mathrm{Z}}$ mass for the combined ${4{\mathrm{e}}}$, ${2{\mathrm{e}} 2{\upmu }}$, and ${4{\upmu }}$ channels. Points represent the data with error bars showing the statistical uncertainties, the shaded histograms represent the SM prediction including signal and irreducible background from simulation, and the reducible background estimate from data. Dashed histogram represents an example of the aTGC signal. The last bin includes contribution from all events with mass above 1300$\,\text {GeV}$

The total ${{\mathrm{Z}} {\mathrm{Z}} }$ cross section is shown in Fig. 3 as a function of the ${\mathrm{p}} {\mathrm{p}}$ center-of-mass energy. Results from CMS [4, 5] and ATLAS [9, 10, 14] are compared to predictions from Matrix  v2.0.0_beta1 and mcfm. The uncertainties are statistical (inner bars) and statistical and systematic combined, as obtained from the fit (outer bars). The band around the Matrix predictions reflects scale uncertainties, while the band around the mcfm predictions reflects both scale and PDF uncertainties.

## Differential cross sections

The differential distributions normalized to the fiducial cross sections are presented in Figs. 4, 5 and 6 for the combination of the ${4{\mathrm{e}}}$, ${2{\mathrm{e}} 2{\upmu }}$, and ${4{\upmu }}$ decay channels using the whole data sample. The fiducial cross section definition includes ${p_{\mathrm {T}} ^{\ell }}$ and ${|\eta ^{\ell } |}$ selections on each lepton, and the 60–120$\,\text {GeV}$ mass requirement, as described in Sect. 4. Figure 4 shows the differential cross sections in bins of $p_{\mathrm {T}}$ for: (left) all leptons in the event, (right) both ${{\mathrm{Z}}}$ bosons in the event, and in Fig. 5 (left) for the $p_{\mathrm {T}}$ of the ${{\mathrm{Z}} {\mathrm{Z}} }$ system. Figure 5 (right) shows the normalized ${\mathrm{d}\sigma /\mathrm{d}m_{{{\mathrm{Z}} {\mathrm{Z}} }}}$ distribution. All $p_{\mathrm {T}}$ and ${m_{{\mathrm{Z}} {\mathrm{Z}} }}$ distributions include overflow in the last bin. Figure 6 shows the angular correlations between ${{\mathrm{Z}}}$ bosons. The data are corrected for background contributions and unfolded for detector effects using a matrix inversion method without regularization as described in Ref. [61], and compared with the theoretical predictions from powheg+mcfm, MadGraph 5_amc@nlo+mcfm and Matrix. The distributions include both Z boson candidates or all four leptons, where applicable, and are normalized to the numbers of objects in the event and to the fiducial cross section. The bottom part of each plot shows the ratio of the measured to the predicted values. The bin sizes are chosen according to the resolution of the relevant variables, trying also to keep the statistical uncertainties at a similar level for all the bins.

The distributions predicted by powheg+mcfm and MadGraph 5_amc@nlo+mcfm agree well with data, except for ${m_{{\mathrm{Z}} {\mathrm{Z}} }}$. This distribution shows a small overestimate in the cross section at high invariant masses. The Matrix predictions describe this region better, which can be explained by the presence of the EW corrections in the Matrix calculations. The effect of EW corrections is in detail discussed in Ref. [44] and can reach 20–30% for ${m_{{\mathrm{Z}} {\mathrm{Z}} } = 1 \,\text {TeV}}$. On the other hand, the Matrix predictions show some deviation from the measurements as a function of ${p_{\mathrm {T}} ^{{\mathrm{Z}} {\mathrm{Z}} }}$ and for the azimuthal separation between the two Z bosons, which is not observed for powheg+mcfm and MadGraph 5_amc@nlo+mcfm predictions.

## Limits on anomalous triple gauge couplings

The presence of aTGCs is expected to increase the event yield at high four-lepton masses. Figure 7 presents the distribution of the four-lepton reconstructed mass for the combined ${4{\mathrm{e}}}$, ${2{\mathrm{e}} 2{\upmu }}$, and ${4{\upmu }}$ channels, for the SM and an example of nonzero aTGC value with $f_4^\gamma =0$, and ${f_4^{\mathrm{Z}} =0.0015}$. Limits on aTGCs are derived from fits to this distribution. The shaded histograms represent the SM predictions as described in the previous sections and the dashed curve shows the sherpa prediction. The sherpa SM predictions are normalized to the powheg+mcfm predictions including *K* factors and agree well with them in shape, as shown in Fig. 7. As a cross-check of the procedure, the sherpa SM distribution was also corrected bin-by-bin to the powheg+mcfm distribution, no difference was observed in the extracted limits. The presence of aTGC contribution increases the expected event yields at masses above 1300$\,\text {GeV}$. In the fit, described below, this region is subdivided into two bins: 1300–2000$\,\text {GeV}$ and above 2000$\,\text {GeV}$. Typically 60–70% of the aTGC events have masses above 2000$\,\text {GeV}$, whereas the expected SM contribution is approximately 2.4 and 0.2 events in the 1300–2000$\,\text {GeV}$ and above 2000$\,\text {GeV}$ bins, respectively.

**Fig. 8:**
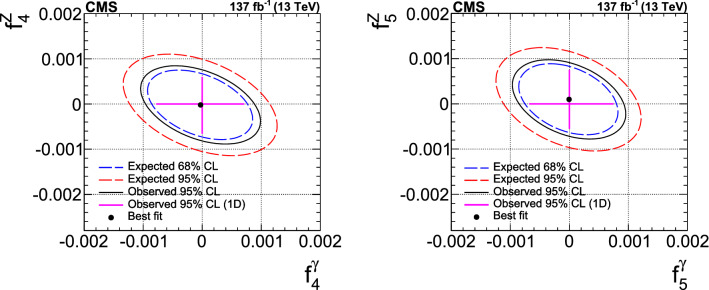
Two-dimensional observed (solid) and expected (dashed) contours exclusion limits at 95% $\text {CL}$, and at 68 and 95% $\text {CL}$, respectively, on the ${{\mathrm{Z}} {\mathrm{Z}} {\mathrm{Z}} }$ and ${{\mathrm{Z}} {\mathrm{Z}} \gamma }$ aTGCs. The plots show the exclusion contours in the ${(f_{4(5)}^{\mathrm{Z}}, f_{4(5)}^\gamma )}$ parameter planes. Dots show where the likelihoods reach their maximum. The coupling values outside the contours are excluded at the corresponding confidence level. The crosses in the middle represent the observed 1D limits. No form factor is used

The invariant mass distributions are interpolated from those obtained from the sherpa simulation for different values of the anomalous couplings in the range between 0 and 0.03. For each distribution, only one or two couplings are varied while all others are set to zero, thus creating a grid of points in the ${(f_{4}^{\mathrm{Z}}, f_{4}^\gamma )}$ and ${(f_{5}^{\mathrm{Z}}, f_{5}^\gamma )}$ parameter planes and the corresponding invariant mass distributions. In each ${m_{{\mathrm{Z}} {\mathrm{Z}} }}$ bin, expected signal values are interpolated between the two-dimensional grid points using a second-order polynomial, since the cross section for the signal depends quadratically on the coupling parameters. A simultaneous fit to the values of aTGCs is performed for all lepton channels, see Ref. [62] for details. A profile likelihood method [53], Wald Gaussian approximation, and Wilks theorem [63] are used to derive one-(1D) and two-dimensional limits at 68 and 95% confidence levels ($\text {CL}$) on each of the aTGC parameters and combination of two of them, while all other parameters are set to their SM values. All systematic uncertainties are included by varying the number of expected signal and background events within their uncertainties. An additional 10% uncertainty is applied on the predictions of the SM and aTGC models to account for possible differences between model predictions and the interpolation used in the fit. No form factor [64] is used when deriving the limits; the results assume that the energy scale of new physics is very high. The constraints on anomalous couplings are displayed in Fig. 8. The curves indicate 68 and 95% $\text {CL}$ contours; the dots indicate where the likelihoods reach their maximum. Coupling values outside the contours are excluded at the corresponding $\text {CL}$. The crosses in the middle represent the observed 1D limits that are summarized in Table 5. The sensitivity is dominated by the statistical uncertainties.

Complete one-loop EW corrections to massive vector boson pair production [66, 67] were applied as a cross-check. The EW corrections to the ${\mathrm{Z}} {\mathrm{Z}}$ production cause the ${\mathrm{Z}} {\mathrm{Z}}$ mass spectrum to fall more rapidly at large masses. In addition, the overall cross section decreases by about 4%. The effect of NLO EW corrections is estimated by reweighting the SM sherpa sample as a function of ${m_{{\mathrm{Z}} {\mathrm{Z}} }}$ using weights derivedfrom the calculations described in Ref. [66]. This reweighting improves the expected limits by about 4–6%, whereas there is no effect on the observed limits. This is expected, since only the SM contribution is subject to the EW corrections; they are not applied on aTGCs. The limits are driven by the high mass tail above 1300 GeV. In this region the aTGC signal is much larger than the SM, and therefore the EW correction on the SM part has a very small effect on the predictions of the SM+aTGC model. This correction is much smaller than the uncertainty we apply in the fit procedure.

These results can be also expressed in terms of EFT parameters. The numerical relations between aTGCs and EFT parameters are given in Ref. [65]. The expected and measured limits in terms of EFT are presented in Table 5.

## Summary

Four-lepton final states have been studied in proton–proton collisions at $\sqrt{s} = 13\,\text {TeV}$ with the CMS detector at the CERN LHC. The data sample corresponds to an integrated luminosity of 137$\,\text {fb}^{-1}$, collected during 2016–2018. Themeasured ${{\mathrm{p}} {\mathrm{p}} \rightarrow {\mathrm{Z}} {\mathrm{Z}} }$ total cross section is $\sigma _{\text {tot}} ( {\mathrm{p}} {\mathrm{p}} \rightarrow {\mathrm{Z}} {\mathrm{Z}} ) = 17.4 \pm 0.3 \,\text {(stat)} \pm 0.5 \,\text {(syst)} \pm 0.4 \,\text {(theo)} \pm 0.3 \,\text {(lumi)} \text { pb}$, where the ${{\mathrm{Z}}}$ boson masses are in the range $60< m_{{\mathrm{Z}}} < 120\,\text {GeV}$. The results agree with the SM predictions, discussed in Sect. 8. The differential cross sections also agree well with the SM predictions. Improved limits on anomalous ${{\mathrm{Z}} {\mathrm{Z}} {\mathrm{Z}}}$ and ${{\mathrm{Z}} {\mathrm{Z}} \gamma }$ triple gauge couplings are established. These are the most stringent limits to date on anomalous ${{\mathrm{Z}} {\mathrm{Z}} {\mathrm{Z}}}$ and ${{\mathrm{Z}} {\mathrm{Z}} \gamma }$ triple gauge couplings and they improve the previous strictest results from CMS by $\approx 30$–40%.

**Table 5:** Expected and observed one-dimensional 95% $\text {CL}$ limits on aTGC parameters. The corresponding constrains on EFT parameters are estimated using the transformation from Ref. [65]

	Expected 95% $\text {CL}$	Observed 95% $\text {CL}$
aTGC parameter	$\times 10^{-4}$	$\times 10^{-4}$
${f_4^{\mathrm{Z}}}$	$-$8.8 ; 8.3	$-$6.6 ; 6.0
${f_5^{\mathrm{Z}}}$	$-$8.0 ; 9.9	$-$5.5 ; 7.5
$f_4^{\gamma }$	$-$9.9 ; 9.5	$-$7.8 ; 7.1
$f_5^{\gamma }$	$-$9.2 ; 9.8	$-$6.8 ; 7.5
EFT parameter	$\text {TeV} ^{-4}$	$\text {TeV} ^{-4}$
${C_{\mathrm {\tilde{B}}{\mathrm{W}}}/\varLambda ^4}$	$-$3.1 ; 3.3	$-$2.3 ; 2.5
${C_{{\mathrm{W}} {\mathrm{W}}}/\varLambda ^4}$	$-$1.7 ; 1.6	$-$1.4 ; 1.2
${C_{\mathrm {B}{\mathrm{W}}}/\varLambda ^4}$	$-$1.8 ; 1.9	$-$1.4 ; 1.3
$C_{\mathrm {BB}}/\varLambda ^4$	$-$1.6 ; 1.6	$-$1.2 ; 1.2

## Data Availability

This manuscript has no associated data or the data will not be deposited. [Authors’ comment: Release and preservation of data used by the CMS Collaboration as the basis for publications is guided by the CMS policy as written in its document “CMS data preservation, re-use and open access policy” (https://cms-docdb.cern.ch/cgi-bin/PublicDocDB/RetrieveFile?docid=6032filename=CMSDataPolicyV1.2.pdf&version=2)].
